# A network meta-analysis on the improvement of cognition in patients with vascular dementia by different acupuncture therapies

**DOI:** 10.3389/fnins.2022.1053283

**Published:** 2022-12-14

**Authors:** Jiayu Wen, Yu Cao, Surui Chang, Qiaoyi Huang, Zhen Zhang, Wei Wei, Jiuxiu Yao, Hui Pei, Hao Li

**Affiliations:** ^1^Graduate College, Beijing University of Chinese Medicine, Beijing, China; ^2^Department of Geriatrics, Xiyuan Hospital, China Academy of Chinese Medical Sciences, Beijing, China; ^3^Wangjing Hospital, China Academy of Chinese Medical Sciences, Beijing, China; ^4^First Clinical College, Shandong University of Traditional Chinese Medicine, Jinan, China

**Keywords:** vascular dementia, electroacupuncture, scalp acupuncture, body acupuncture, moxibustion, cognitive function, ability of daily life, frequentist network meta-analysis

## Abstract

**Introduction:**

The second most prevalent cause of dementia is vascular dementia (VaD). Furthermore, acupuncture is a relatively safe and effective traditional therapy for individuals with VaD. We performed a network meta-analysis to assess the effectiveness and safety of various acupuncture therapies for VaD based on existing research.

**Methods:**

We searched six electronic databases to screen for randomized controlled trials (RCTs) comparing different acupuncture treatments in VaD patients. The Cochrne tool (Review Manager 5.3) was used to evaluate the risk of bias of the included RCTs. Based on the Grading of Recommendations Assessment, Development and Evaluation framework, we assessed the confidence in the evidence using the Confidence In the results from Network Meta-Analysis approach. We used the frequency approach to perform the network meta-analysis. Data were analyzed using R 4.1.1.

**Results:**

In total, we included 46 eligible studies. The results of the network analysis showed that the combined interventions of moxibustion (MB) with body acupuncture (BA) (MB + BA) and electroacupuncture (EA) with scalp acupuncture (SA) with BA (EA + SA + BA) were more effective in improving cognitive functions and activities of daily living compared with SA or BA alone. However, in the subgroup analysis, EA + SA + BA showed better efficacy in short- and mid-term acupuncture compared with other acupuncture therapies.

**Conclusion:**

Combined acupuncture therapy may be a safe and effective intervention for individuals with VaD, and MB + BA and EA + SA + BA appear to be the most effective interventions. However, because the analysis of this study was based on low-to-moderate evidence, there remains no strong supporting evidence. Thus, high-quality, large-scale, and long-term studies should be conducted in the future to assess the effectiveness and safety of acupuncture in VaD.

**Systematic review registration:**

https://www.crd.york.ac.uk/prospero/, identifier: CRD42022354573.

## Introduction

Vascular dementia (VaD) is the second most prevalent cause of dementia (Yang et al., 2022), accounting for roughly 15% to 20% of dementia cases in North America and Europe, and about 30% of dementia cases in Asia (Wolters and Ikram, 2019). The risk of VaD doubles every 5.3 years, with the incidence rate increasing with age. Age-related dementia has become one of the major health problems worldwide (Iadecola et al., 2019). According to the World Health Organization, 35.6 million individuals worldwide suffer from dementia, and this figure is anticipated to quadruple by 2050 (World Health, 2012), which would place a huge burden on families, primary caregivers, and the economic cost to society. In the United States, such costs have exceeded those of cancer and heart disease (Hurd et al., 2013). Although the average cognitive impairment rates of VaD and Alzheimer's disease are similar, the mortality rate is higher in VaD than that in Alzheimer's disease (Kua et al., 2014). Therefore, there is an urgent need to identify suitable treatments for VaD.

VaD is characterized by progressive deterioration of memory and other cognitive functions due to cerebrovascular disease. The major cause of VaD is chronic cerebral hypoperfusion (Wang et al., 2020) and tissue hypoxia (Iadecola et al., 2019) after cerebral ischemia, which enhances the permeability of the blood-brain barrier permeability and plasma protein extravasation into the brain, resulting in a severe inflammatory response and oxidative stress, further leading to white matter damage (Rouhl et al., 2012). Although several advancements have been made in understanding the relationship between cerebrovascular disease and dementia, the underlying pathogenesis of VaD remains poorly understood. Currently, beneficial therapies include cholinesterase inhibitors and memantine, which are licensed drugs for Alzheimer's disease (O'Brien and Thomas, 2015). Several large studies have shown that cholinesterase inhibitors and memantine are effective in the treatment of VaD; however, the magnitude of their effectiveness is limited. Therefore, the regulators and guideline panels of these trials have recommended that memantine and cholinesterase inhibitors are inappropriate for the treatment of patients with VaD (Joshua et al., 2018). The same phenomenon is observed with N-methyl-D-aspartate antagonists (Orgogozo et al., 2002; Wilcock et al., 2002). For these reasons, alternative approaches, including acupuncture, have been adopted for VaD.

Acupuncture, which is a traditional Chinese medical therapy, is one of the most commonly employed non-pharmacological therapies, and has been used in many countries for the treatment of neurological disorders (Wu et al., 2010; Wan et al., 2016). Accumulated evidence has also shown that acupuncture can improve the symptoms of VaD through its antioxidant, anti-inflammatory, and anti-apoptotic effects (Ye et al., 2017; Yang et al., 2018; Zhu et al., 2018). The network meta-analysis (NMA) method is effective for comparing and ranking various therapies; therefore, this study aimed to assess the efficacy and safety of different acupuncture techniques for the treatment of patients with VaD, and to determine the most suitable method for the acupuncture treatment of VaD.

## Methods

This review was conducted according to the Preferred Reporting Items for Systematic Reviews and Meta-Analyses for Network Meta-Analyses (Moher et al., 2009; Page et al., 2021). In addition, our study protocol has been registered with the International Prospective Register of Systematic Reviews (PROSPERO) (registration number: CRD42022354573).

### Search strategy for identification of studies

We conducted a systematic search of six databases, including PubMed, Cochrane Library, Embase, Web of Science, MEDLINE, and China National Knowledge Infrastructure, from their establishment on July 31, 2022. The following keywords were used during the search: (acupuncture therapy, acupuncture, acupuncture treatment, electro-acupuncture, needling, scalp acupuncture, electrostimulation, body acupuncture, electroacupuncture, moxibustion) and (dementia, vascular dementia, infarct dementia, post-stroke dementia, and vascular cognitive impairment). We adjusted and specified the retrieval strategies according to the different databases. The specifics of the search strategies are listed in the Supplementary Appendix 1.

### Study selection

Two reviewers (JYW and SRC) independently evaluated the included studies. Any discrepancies were reviewed by a third reviewer (ZZ) and resolved by discussion among all the reviewers. Included studies fulfilled the following criteria: (i) randomized controlled trials (RCTs); (ii) included patients who matched the established diagnostic criteria for VaD, including the Diagnostic and Statistical Manual of Mental Disorders, the National Institute of Neurological Disorders and Stroke, and the Association Internationale pour la Recherche et l'Enseignement en Neurosciences criteria, Hachinski Ischemic Score ≥ 7, and the outcomes of computed tomography or magnetic resonance imaging; (iii) types of acupuncture included were electroacupuncture (EA), scalp acupuncture (SA) (including traditional SA and modern SA) (Jian-Li et al., 2019), body acupuncture (BA), and moxibustion (MB); and (iv) the control group received sham acupuncture, blank control, wait-list control and anti-dementia medications (for whom the observation group used acupuncture combined with anti-dementia medications). Studies that met the following criteria were excluded: (i) comprised of patients with Alzheimer's disease or dementia caused by other factors; (ii) included patients with Hachinski Ischemic Score < 7; (iii) included patients with a score > 8 on the Cornell Scale for Depression in Dementia and who were diagnosed according to the Diagnostic and Statistical Manual of Mental Disorders criteria as having evident mental depression, or patients with other mental diseases or disorders; and (iv) included patients with severe neurological deficits or serious medical conditions, such as dysopia, aphasia and dysacusis, and malignancy.

### Data abstraction

Two reviewers (JYW and QYH) independently extracted data from the included RCTs. The extracted data included study characteristics such as first author, title of study, participants (sex, age, month of diagnoses, sample sizes), study design (randomization, blinding), interventions, controlled interventions, outcome measures, results and adverse events, we recorded these characteristics in advance. The four categories of acupuncture treatments were EA, SA, BA, and MB. The control groups included patients receiving anti-dementia medications, for whom the observation group used acupuncture combined with anti-dementia medications; sham acupuncture; blank control; wait-list control. Any discrepancies were evaluated by a third reviewer (JXY) and resolved by discussion among all the reviewers.

### Outcomes

The Mini-Mental State Examination (MMSE) and Hasegawa Dementia Scale (HDS) were defined as the primary efficiency outcome measures. When more than one measure of cognitive function was used in a study, we preferentially used the MMSE in all cases for inclusion in our meta-analysis. However, due to the high similarity in scoring criteria, total score and content between MMSE and HDS (Kim et al., 2005; Senda et al., 2020), we included a very small number of HDS scores to analyze together. The Barthel Index of ADLs and activities of daily living (ADLs) were identified as the secondary efficacy outcome indicators.

### Quality assessment

Two independent reviewers (SRC and WW) evaluated the identified trials. The revised Cochrane risk-of-bias tool for randomized trials (Review Manager 5.3) was used to assess the bias risk of the included RCTs. Any disagreements were reviewed by a third reviewer (QYH) and resolved by discussion among all the reviewers.

### Data synthesis and analysis

A pairwise meta-analysis was performed in this study using the random-effects model in Stata 14 (StataCorp LLC, College Station, Texas 77845, USA). Then, the “netmeta” version 0.9–2 of the R-4.1.1 software was used to conduct a frequentist NMA (Rücker, 2012; Krahn et al., 2013) in this study. To display and describe the geometric features of different acupuncture treatments, we used the “networkPlot” function of Stata 14 (College Station, Texas 77,845 USA) to draw and generate network graphs. Different nodes represented different treatments, and edges represented head-to-head comparisons between different treatments. We used the “decomp.design” function to evaluate the consistency of entire network, homogeneity within designs, and homogeneity/consistency between designs. We used a node-splitting method (Rücker and Schwarzer, 2015) to evaluate the inconsistency between direct and indirect comparisons. The different interventions were ranked in accordance with the P-score, which were based only on the standard errors of the network and point estimations. These scores, which gauge the degree of certainty between comparisons of different interventions, are averaged across all competing treatments (Brehm et al., 2009).

We calculated pooled estimates and 95% confidence intervals (95% CIs) by performing a random-effects NMA. Generally speaking, when studies use the same evaluation method to assess our results of interest, the mean difference in the change scores between the final and baseline scores on the scale was regarded as a treatment effect to evaluate the outcomes. Since lower scores represented more severe cognitive impairment, the final minus the baseline scores served as the change scores definition. One treatment was considered more effective than the other if the corresponding estimate of the mean difference in the change score was positive and the 95% CI did not include zero.

Moreover, to investigate the probable sources of heterogeneity more thoroughly in this study, we performed subgroup analyses according to different acupuncture treatment durations. Based on the duration of treatment, all results from the included studies were divided into three groups i.e., short-term (1 ≤ × ≤ 30 days), mid-term (30 < × ≤ 60 days), and long-term (× > 60 days).

### Assessing confidence of the evidence

Confidence in the results from Network Meta-Analysis (CINeMA) was used to assess the confidence of the evidence in all the trials, and ratings were assigned using the CINeMA application (Nikolakopoulou et al., 2020).

CINeMA evaluates six domains as follows: within-study bias, reporting bias, indirectness, imprecision, heterogeneity, and incoherence. With the exception of reporting bias, which is classified as suspect or undetected, they are graded as no concerns, some concerns, or major concerns. The conclusions are then presented for each treatment comparison across these six dimensions as high, moderate, low, or very low confidence (Papakonstantinou et al., 2020).

All or most comparisons from industry-funded trials were considered to be at a risk of reporting bias. For comparisons with poor network connections, indirection is downgraded. For imprecision, the threshold was set to a mean difference of 0 for continuous comparisons.

## Results

### Study identification

**Figure 1:**
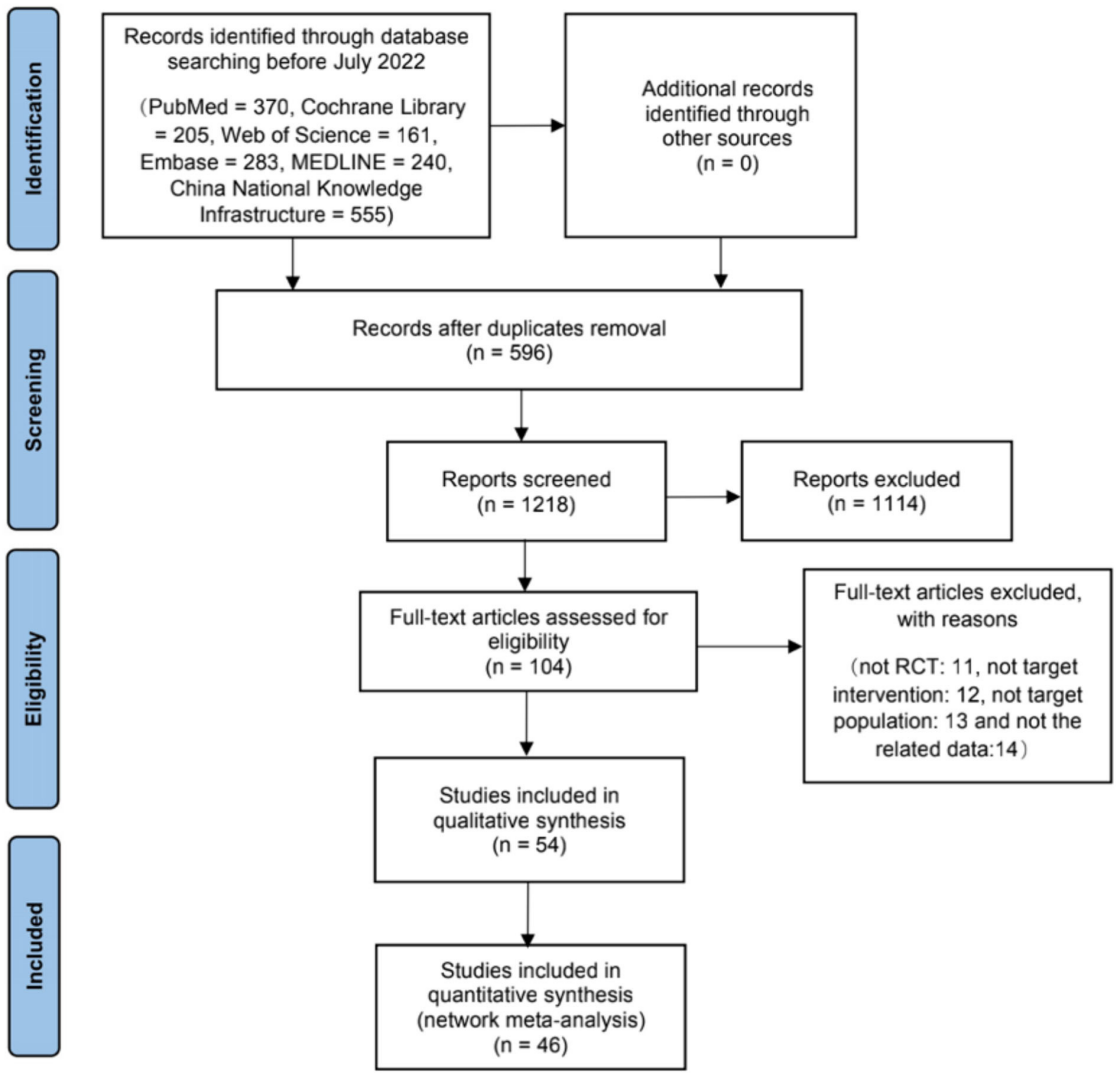
Flowchart of literature selection on systematic reviews on acupuncture for treating vascular dementia.

For the present study, a total of 1,814 studies were identified. After reviewing the titles and abstracts, 102 studies were chosen for further review. Of these, 50 were excluded: 14 did not report the related data, 13 did not include the relevant target populations, 11 were not RCTs, and 12 did not investigate the target interventions. After qualitative synthesis, 8 studies were excluded. In total, 46 RCTs met the inclusion criteria and were included in this meta-analysis study (Huang et al., 1992, 2007, 2012; Liu et al., 1997, 1998, 2016; Lai et al., 1998; Li et al., 1999, 2014; Lun et al., 2003; Lai and Huang, 2005; Wu, 2006; Xu, 2006; Zhang et al., 2006, 2008; Ling et al., 2007; Chu et al., 2008; Han, 2009; Peng, 2009; Wang, 2009, 2014; Zhao et al., 2009; Teng, 2011; Yin et al., 2011; Teng and Lai, 2012; Zhou and Zhou, 2012; Dai et al., 2013a,b; Gao et al., 2013; Sheng et al., 2013; He and Guo, 2014; Shi et al., 2014; Jin, 2015; Li, 2015; Luo et al., 2015; Xie and Junming, 2016; Chen, 2017; Sheng and Cai, 2017; Wang and Wang, 2018; Wang et al., 2018; Feng, 2020; Guo, 2020; Ouyang, 2020; Yao, 2020; Zhu, 2020; Han et al., 2021). The detailed selection process is illustrated in Figure 1.

### Characteristics of the included studies

The aggregated characteristics of the included RCTs are shown in Table 1.

**Table 1:** The aggregated characteristics of the included RCTs.

**Source**	**Study design**	**Age**	**Sample (men)**	**Duration**	**Intervention scheme**	**Duration of intervention**	**Outcome measure**	
Wang et al. (2018)	Randomized controlled trial	SA: 70.0 ± 8.0 Control: 64.0 ± 7.0	SA: 60 (31) Control: 60 (32)	SA: 8.3 ± 3.3 Control: 8.2 ± 2.7 years	SA: 30 min, manipulate the needle for 60 s	2 times per day, 5 times per week for 7 weeks	MMSE	N/A
Han et al. (2021)	Randomized controlled trial	SA + BA: 64.0 ± 9.0 Control: 66.0 ± 8.0	SA: 59 (30) Control: 59 (29)	SA: 6.3 ± 2.6 Control: 6.7 ± 2.4 months	SA: 30 min, manipulate the needle until patients have the needling sensation	1 time per day, 5 times per week for 8 weeks	MMSE	Barthel
Guang-Xia et al. (2014)	Randomized controlled trial	SA + BA: 67.2 ± 9.3 Control: 67.5 ± 10.1	SA + BA: 22 (12) Control: 22 (11)	N/A	SA + BA: 30 min, manipulate the needle until patients have the needling sensation	1 time every two days for 6 weeks	MMSE	ADL
Liu et al. (2016)	Randomized controlled trial	SA + BA: 55.0 ± 7.0 Control: 56.0 ± 9.0	SA + BA:84 (54) Control: 84 (52)	SA + BA: 2.9 ± 0.7 Control: 3.2 ± 0.9 years	SA + BA: manipulate the needle for 1 min	1 time per day for 8 weeks	MMSE	N/A
Li et al. (1999)	Randomized controlled trial	58.0–82.0	32 (9)	3–60 years	SA + BA: 30 min	1 time per day for 2 months	HDS	N/A
Huang et al. (1992)	Randomized controlled trial	53.0–75.0	36 (27)	3–60 years	SA+BA	1 month	HDS	N/A
Jin (2015)	Randomized controlled trial	BA: 73.2 ± 5.3 Control: 71.9 ± 4.8	BA: 23 (15) Control: 23 (14)	BA: 2.4 ± 1.3 Control: 2.0 ± 1.7 years	BA: manipulate the needle for 1 min	1 time per day, 6 times per week for 2 months	MMSE	ADL
Li (2015)	Randomized controlled trial	SA + BA: (man) 50.3 ± 3.1, (woman) 51.5 ± 2.5 Control: (man) 53.3 ± 2.5, (woman) 50.6 ±3. 6	SA + BA: 43 (25) Control: 43 (26)	SA + BA: (man) 2.0 ± 1.5, (woman) 2.3 ± 0.9 Control: (man) 2.1 ± 0.5, (woman) 2.0 ± 1.6 years	SA + BA: 40 min, manipulate the needle 1 time	1 time per day, 5 times per week for 12 weeks	MMSE	ADL
Lun et al. (2003)	Randomized controlled trial	SA: 52.0–68.0 Control: 52.0–65.0	SA: 57 (35) Control: 32 (20)	N/A	SA: 30 min, manipulate the needle 2 times	1 time per day, 6 times per week for 4 months	HDS	N/A
Wang et al. (2018)	Randomized controlled trial	MB + BA: 54.0 ± 7 0.0 MB: 52.0 ± 7.0 BA: 52.0 ± 8.0	MB + BA: 38 (28) MB: 38 (29) BA: 38 (27)	MB + BA: 16.1 ± 4.7 MB: 15.9 ± 5.7 BA: 15.8 ± 3.9 months	MB + BA, MB: 30 min BA: 30 min, manipulate the needle until patients have the needling sensation	MB: 1 time per day, 2 times per week for 4 weeks BA: 1 time per day, 5 times per week for 4 weeks	MMSE	ADL
Lai et al. (1998)	Randomized controlled trial	EA + SA + BA: 68.6 ± 6.9 SA + BA: 66.1 ± 6.8	EA + SA + BA: 23 (14) SA + BA: 23 (17)	N/A	EA + SA + BA: 30 min, the frequency of EA is 120–250 times per minute; SA+BA: 30 min, manipulate the needle 1 time every 10 min	1 time per day, 5 times per week for 7 weeks	HDS	N/A
Zhang et al. (2008)	Randomized controlled trial	EA + SA: 64.7 ± 8.7 Control: 65.0 ± 8.2	EA + SA: 82 (50) Control: 81 (53)	N/A	EA + SA: 30 min, 3–15 Hz, 2–4 mA	1 time per day, 5 times per week for 6 weeks	MMSE	ADL-R
Zhao et al. (2009)	Randomized controlled trial	N/A	N/A	N/A	EA + SA: 30 min, the frequency of EA is 300–500 times per minute	1 time per day, 5 times per week for 6 weeks	MMSE	N/A
Liu et al. (1998)	Randomized controlled trial	EA + SA 1: 67.5 ± 7.2 Control 1: 65.5 ± 6.8 EA + SA 2: 68.2 ± 7.8 control 2: 65.2 ± 7.6	EA + SA 1: 30 (15) Control 1: 60 (34) EA + SA 2: 60 (38) Control 2: 30 (21)	EA + SA 1: 11.5 ± 8.7 Control 1: 11.1 ± 8.1 EA + SA 2: 11.8 ± 4.9 Control 2: 13.0 ± 5.4 months	EA + SA: 30 min, the frequency of EA is 200 times per minute	1 time per day, 5 times per week for 8 weeks	MMSE	ADL
Li et al. (2014)	Randomized controlled trial	SA: 63.0 ± 9.0 BA: 64.0 ± 8.0	SA: 31 (16) BA: 28 (14)	SA: 10.6 ± 3.3 BA: 10.7 ± 3.3 years	SA: 45 min, manipulate the needle until patients have the needling sensation	1 time per day for 6 weeks	MMSE	ADL
Lai and Huang (2005)	Randomized controlled trial	73.1 ± 5.8	50 (23)	2.35 ± 0.92 years	SA + BA, BA: 20 min, manipulate the needle 1 time every 5 min	1 time per day, 5 times per week for 4 weeks	HDS	ADL
Huang et al. (2007)	Randomized controlled trial	SA + BA 1: 71.3 ± 4.1 SA + BA 2: 71.6 ± 5.0 SA + BA 3: 69.3 ± 4.7 BA: 71.2 ± 4.8	SA + BA 1: 10 (6) SA + BA 2: 10 (5) SA + BA 3: 10 (5) BA: 10 (4)	SA + BA 1: 2.1 ± 0.7 SA + BA 2: 2.6 ± 0.5 SA + BA 3: 2.2 ± 0.6 BA: 2.2 ± 0.6 years	SA + BA, BA: 20 min, manipulate the needle 1 time every 5 min	1 time per day, 5 times per week for 4 weeks	MMSE	ADL
Liu et al. (1997)	Randomized controlled trial	BA: 64.8 ± 7.3 SA + BA: 64.7 ± 7.4	BA: 50 (40) SA + BA: 51 (33)	BA: 0.5–60 SA + BA: 0.5–36 months	BA, SA + BA: 20 min, manipulate the needle until patients have the needling sensation	1 time per day for 1 month	HDS	N/A
Teng (2011)	Randomized controlled trial	Control: 69.0 ± 6.0 EA + SA: 66.0 ± 6.0 BA: 68.0 ± 6.0	Control: 28 (18) EA + SA: 27 (16) BA: 25 (14)	Control: 2.0 ± 1.1 EA + SA: 2.1 ± 0.7 BA: 2.0 ± 1.1 years	EA + SA, BA: 30 min	1 time per day, 6 times per week for 1 month	MMSE	Barthel
Xu (2006)	Randomized controlled trial	EA + SA: 63.7 ± 7.7 Control: 63.3 ± 8.1	EA + SA: 30 (21) Control: 20 (12)	N/A	EA + SA: 30 min, dense-sparse waves	1 time per day for 3 months	MMSE	ADL
Yao (2020)	Randomized controlled trial	EA + SA: 64.5 ± 4.9 Control: 63.3 ± 5.5	EA + SA: 30 (15) Control: 30 (16)	EA + SA: 26.1 ± 6.5 Control: 25.9 ± 6.0 months	EA + SA: 30 min, continuous wave, 4 Hz	1 time per day for 4 weeks	MMSE	ADL
Wang (2014)	Randomized controlled trial	EA + SA + BA: 66.0 ± 7.9 SA + BA: 63.6 ± 8.0	EA + SA + BA: 30 (16) SA + BA: 30 (17)	EA + SA + BA: 61.6 ± 37.4 SA + BA: 59.6 ± 32.9 months	EA: 20 min, dense-sparse waves SA + BA: 40 min, manipulate the needle 1 time every 20 min	1 time per day, 5 times per week for 8 weeks	MMSE	ADL
Ling et al. (2007)	Randomized controlled trial	N/A	N/A	N/A	EA + SA: 30 min, continuous wave, the frequency of EA is 300–500 times per minute	1 time per day, 5 times per week for 6 weeks	MMSE	N/A
Yin et al. (2011)	Randomized controlled trial	EA + SA: 62.7 ± 5.1 Control: 62.8 ± 5.4	EA + SA: 30 (18) Control: 30 (17)	EA + SA: 1.7 ± 0.3 Control: 1.6 ± 0.3 years	EA + SA: 30 min, continuous wave, 25–42 Hz	1 time per day, 5 times per week for 12 weeks	MMSE	N/A
Zhang et al. (2006)	Randomized controlled trial	EA + SA: 67.6 ± 7.0 Control: 63.0 ± 6.7	EA + SA: 27 (19) Control: 28 (17)	N/A	EA + SA: 30 min, continuous wave, the frequency of EA is 300–500 times per minute	1 time per day, 5 times per week for 6 weeks	MMSE	ADL
Han (2009)	Randomized controlled trial	EA + SA + BA: 62.5 ± 15.8 Control: 64.2 ± 16.3	EA + SA + BA: 92 (49) Control: 90 (47)	N/A	EA + SA + BA: 30 min, dense-sparse waves	1 time per day, 5 times per week for 8 weeks	LOCTA	N/A
Zhu (2020)	Randomized controlled trial	EA + SA + BA: 65.8 ± 6.7 SA + BA: 65.6 ± 7.2	EA + SA + BA: 32 (15) SA + BA: 31 (16)	EA + SA + BA: 12.6 ± 3.4 SA + BA: 12.2 ± 3.2 months	EA + SA + BA: 30 min, continuous wave, 2 Hz SA + BA: 30 min, manipulate the needle for 1 minute	2 time per day, 6 times per week for 4 weeks	MMSE	ADL
Peng (2009)	Randomized controlled trial	EA + SA: 63.7 ± 9.3 Control: 66.0 ±9 0.1	EA + SA: 24 (15) Control :26 (17)	N/A	EA + SA: 30 min, continuous wave, the frequency of EA is 300–500 times per minute	1 time per day, 5 times per week for 6 weeks	MMSE	ADL
Guo (2020)	Randomized controlled trial	EA + SA + BA: 67.4 ± 3.4 SA + BA: 66.7 ± 3.4	EA + SA + BA: 30 (13) SA + BA: 30 (16)	N/A	EA + SA + BA: 30 min, dense-sparse waves, manipulate the needle for 1 min	1 time per day, 6 times per week for 5 weeks	MMSE	N/A
Chu et al. (2008)	Randomized controlled trial	SA: 68.2 ± 9.2 Control: 67.7 ± 8.5	SA: 33 (20) Control: 32 (18)	SA: 5.2 ± 2.9 Control: 4.9 ± 2.8 months	SA: 10 h, manipulate the needle until patients have the needling sensation	1 time per day, 5 times per week for 8 weeks	MMSE	ADL
Chen (2017)	Randomized controlled trial	EA + SA: 58.1 ± 6.9 Control: 57.4 ± 6.7	EA + SA: 37 (20) Control: 37 (21)	EA + SA: 13.5 ± 4.7 Control: 13.5 ± 4.7 months	EA + SA: 30 min SA: 6 h	1 time per day, 6 times per week for 6 weeks	MMSE	N/A
Wu (2006)	Randomized controlled trial	64.7 ± 7.6	60 (38)	N/A	EA + SA: 20 min, the frequency of EA is 200 times per minute	1 time per day for 45 days	MMSE	N/A
Zhou and Zhou (2012)	Randomized controlled trial	SA + BA: 64.8 ± 6.2 BA: 66.3 ± 7.3	SA + BA: 30 (18) BA: 30 (20)	N/A	SA + BA, BA: 30 min, manipulate the needle 1 time every 10 min	1 time per day, 6 times per week for 6 weeks	MMSE	ADL
Sheng et al. (2013)	Randomized controlled trial	SA: 71.8 ± 2.7 Control: 67.3 ± 2.0	SA: 30 (22) Control: 30 (19)	SA: 10.4 ± 2.3 Control: 10.6 ± 2.3 months	SA: 30 min, manipulate the needle until patients have the needling sensation	1 time per day, 6 times per week for 12 weeks	MMSE	ADL
Dai et al. (2013b)	Randomized controlled trial	N/A	N/A	N/A	SA: 50 min, manipulate the needle 2 times, 2 min every time	1 time per day for 4 weeks	MMSE	ADL
Dai et al. (2013a)	Randomized controlled trial	SA 1: 66.0 ± 6.0 SA 2: 68 ± 6.0 Control: 69.0 ± 6.0	SA 1: 30 (17) SA 2: 30 (15) Control: 30 (10)	SA 1: 2.1 ± 0.7 SA 2: 2.0 ± 1.0 Control: 2.0 ± 1.0 years	SA 1, SA 2: 50 min, manipulate the needle 2 times, 2 min every time	1 time per day for 4 weeks	MMSE	ADL
He and Guo (2014)	Randomized controlled trial	EA + SA: 65.1 ± 8.5 BA: 66.4 ± 7.2	SA: 34 (18) BA: 33 (17)	SA: 9.8 ± 2.9 BA: 9.9 ± 2.9 years	SA, BA: 50 min, manipulate the needle until patients have the needling sensation	1 time per day for 8 weeks	MMSE	ADL
Gao et al. (2013)	Randomized controlled trial	SA: 71.6 ± 5.0 Control: 72.3 ± 4.0	EA + SA: 30 (13) Control: 30 (14)	EA + SA: 5.6 ± 2.1 Control: 5.0 ± 1.9 months	EA + SA: 30 min, continuous wave, the frequency of EA is 300–500 times per minute	1 time per day, 6 times per week for 8 weeks	MMSE	N/A
Teng and Lai (2012)	Randomized controlled trial	SA: 66.0 ± 6.0 BA: 68.0 ± 6.0	SA: 27 (16) BA: 25 (14)	SA: 2.1 ± 0.7 BA: 2.0 ± 1.1 years	SA: 30 min	1 time per day, 6 times per week for 1 month	MMSE	Barthel
Wang (2009)	Randomized controlled trial	EA + SA: 66.8 ± 8.6 EA + BA: 66.2 ± 8.1	EA + SA: 92 (51) EA + BA: 92 (59)	EA + SA: 3.6 ± 2.9 EA + BA: 3.6 ± 2.9 years	EA + SA, EA + BA: 30 min, dense-sparse waves, 80–100 Hz	1 time per day, 5 times per week for 3 months	MMSE	ADL
Huang et al. (2012)	Randomized controlled trial	EA + SA: 67.0 ± 9.0 EA + BA: 66.0 ± 8.0	EA + SA: 92 (51) EA + BA: 92 (59)	N/A	EA + SA, EA + BA: 30 min, dense-sparse waves, 1.3–1.7 Hz	1 time per day, 5 times per week for 3 months	MMSE	ADL
Xie and Junming (2016)	Randomized controlled trial	SA: 63.5 ± 7.8 Control: 62.8 ± 7.1	SA: 30 (17) Control: 30 (16)	SA: 2.7 ± 0. 5 Control: 2.9 ± 0. 3 years	SA: 45 min, manipulate the needle 2 times every 10 min, 10–15 s per time	1 time per day for 3 months	MMSE	ADL
Ouyang (2020)	Randomized controlled trial	SA + BA: 63.8 ± 9.4 Control: 62.6 ± 9.6	SA + BA: 30 (12) Control: 30 (11)	SA + BA: 1.3 ± 0.7 Control: 1.4 ± 0.7 years	SA + BA: 30 min, manipulate the needle until patients have the needling sensation	1 time per day, 6 times per week for 4 weeks	MMSE	ADL
Feng (2020)	Randomized controlled trial	EA + SA: 65. 8± 5.3 Control: 64.3 ± 6.0	EA + SA: 30 (17) Control: 30 (16)	EA + SA: 14.3 ± 2.5 Control: 14.7 ± 1.7 months	EA + SA: 30 min, dense-sparse waves, 1 Hz	1 time per day, 5 times per week for 8 weeks	MMSE	ADL
Luo et al. (2015)	Randomized controlled trial	MB: 72.1 ± 3.4 BA: 71.8 ± 3. 9	MB: 30 (13) BA: 30 (15)	MB: 1.4 ± 0.3 BA: 1.4 ± 0.2 years	MB: 30 min BA: 30 min, manipulate the needle for 30 s	1 time every two days for 8 weeks	MMSE	N/A
Sheng and Cai (2017)	Randomized controlled trial	MB: 55.5 ± 4.9 Control: 54.9 ± 5.2	MB: 30 (16) Control: 30 (18)	N/A	MB: 30 min	1 time per day, 6 times per week for 4 weeks	MMSE	ADL

The included studies were released between 1992 and 2022. Forty-six of the RCTs originated in China. Thirty-seven studies were published in Chinese, whereas nine studies were published in English. Thirty RCTs were two-arm trials, 13 were three-arm trials, and three were multi-arm trials. Courses of acupuncture and other therapies ranged from 4 to 12 weeks in the included studies.

### Quality of evidence

**Figure 2:**
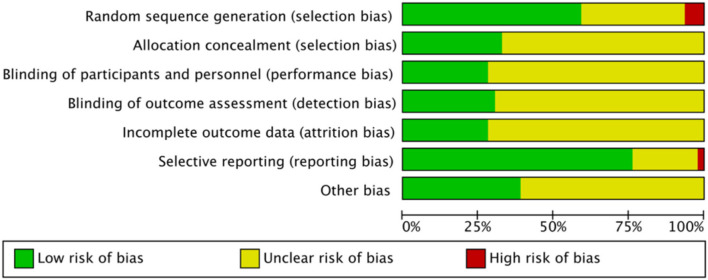
Risk of bias summery for 46 included studies.

Figure 2 presents the evaluation results of the risk of bias (Supplementary Appendix 2). All 46 included studies were RCTs. Methods for generating random sequences were clearly stated in 30 studies, of which 3 studies (Liu et al., 1997; Sheng and Cai, 2017; Wang et al., 2018) were classified as high risk of bias due to the use of hospital or clinical record numbers as sequence numbers. The remaining 16 studies demonstrated an unclear risk of bias, as the random sequence generation method was not specified. Fifteen studies demonstrated a low risk of bias and the remaining 31 studies showed an unclear risk of bias in allocation concealment.

Due to the specificity of the interventions evaluated in this review, blinding between patients and clinicians was difficult to implement. Thirteen studies showed a low risk of bias and 33 studies showed an unclear risk of bias in the blinding of the participants and personnel. Fourteen studies showed a low risk of bias and 32 studies showed an unclear risk of bias in the blinding of outcome assessment.

Thirteen studies showed a low risk of bias in incomplete outcome data, whilst 33 studies showed an unclear risk of bias for incomplete outcome data. Regarding selective outcome reporting, one study (Guang-Xia et al., 2014) demonstrated a high risk of bias because it did not report pre-determined outcomes, 35 studies showed a low risk of bias, and the remaining 10 studies showed an unclear risk of bias. Twenty-eight studies showed an unclear risk of bias due to other bias.

### Outcome analysis of cognitive function

#### Pairwise meta-analysis

We performed a pairwise meta-analysis of cognitive function and the results are shown in Supplementary Appendix 3. The results of the pairwise meta-analysis showed that although each acupuncture therapy had certain advantages compared with the control group (among which the advantages of MB + BA and EA + SA were larger), there was no significant difference in any of the comparisons.

#### Network meta-analysis

**Figure 3:**
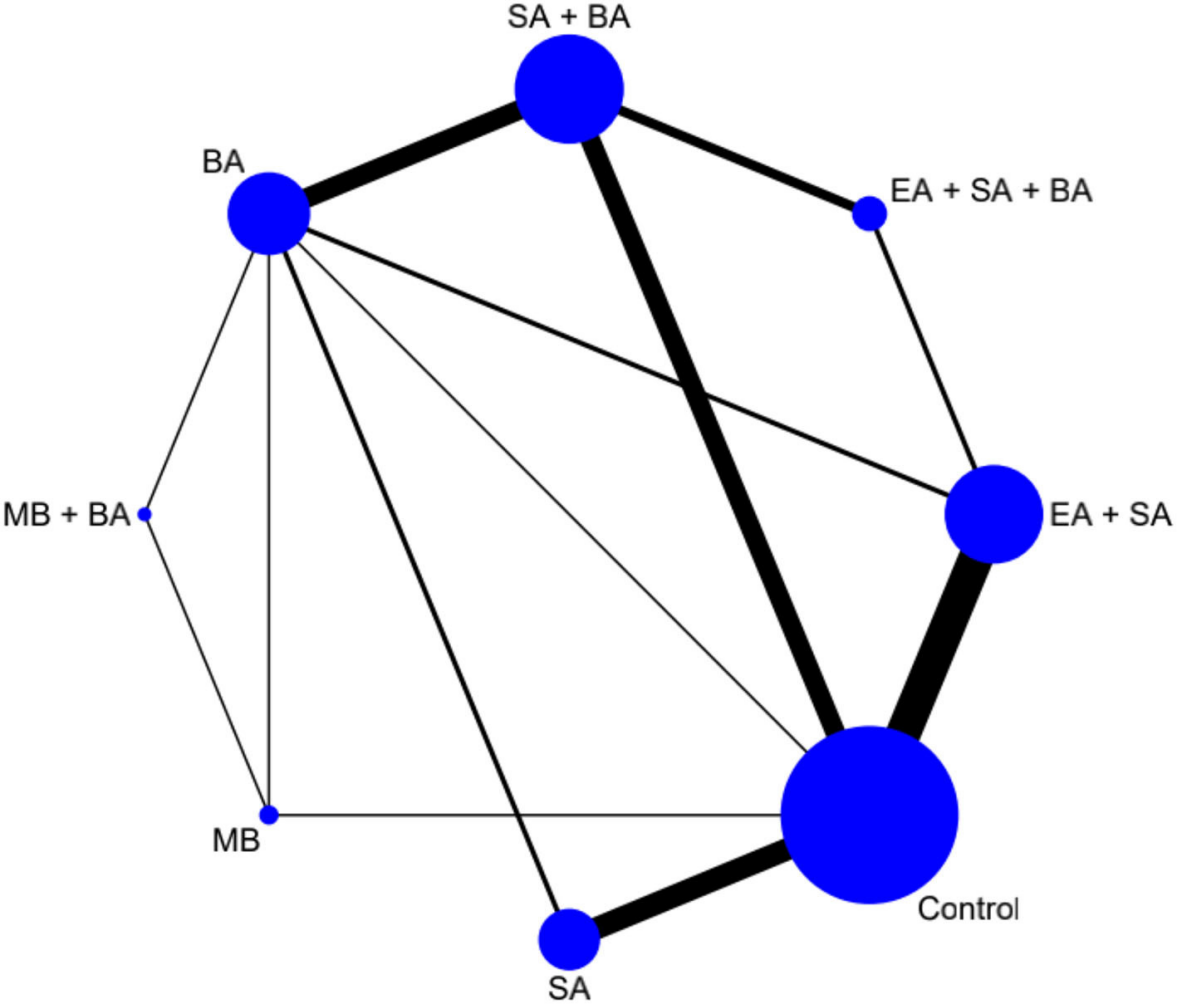
Network plot of cognitive function.

The network plot is shown in Figure 3. Eight interventions were involved i.e., EA + SA + BA, SA + BA, EA + SA, SA, BA, MB + BA, MB, and control.

Forty-six studies, which contributed 3,731 patients in the main NMA, reported changes in cognitive function scores using the MMSE and HDS. The results of the NMA of different interventions are shown in Table 2. Compared to the control group, MB + BA (1.31, 95% CI: 0.18–2.44), EA + SA + BA (1.17, 95% CI: 0.53–1.81), SA + BA (0.92, 95% CI: 0.52–1.33), SA (0.88, 95% CI: 0.45–1.31), EA + SA (0.84, 95% CI: 0.50–1.19), and BA (0.53, 95% CI: 0.06–1.00) showed better improvement in cognitive function.

**Table 2:** Results of network meta-analysis for all possible treatment effects (cognitive function).

**MB + BA**	.	.	.	.	0.61 (−0.77–1.98)	0.68 (−0.70–2.05)	.
0.14 (−1.11–1.40)	**EA + SA + BA**	0.73 (0.03–1.44)	.	−0.56 (−1.50–0.38)	.	.	.
0.39 (−0.75–1.52)	0.24 (−0.35–0.83)	**SA + BA**	.	.	.	0.23 (−0.31–0.76)	1.31 (0.82–1.81)
0.43 (−0.75–1.62)	0.29 (−0.46–1.04)	0.05 (−0.51–0.61)	**SA**	.	.	0.49 (−0.50–1.48)	0.85 (0.38–1.31)
0.47 (−0.70–1.63)	0.32 (−0.32–0.97)	0.08 (−0.40–0.56)	0.03 (−0.51–0.57)	**EA + SA**	.	0.60 (−0.39–1.59)	0.66 (0.29–1.04)
0.50 (−0.57–1.57)	0.36 (−0.73–1.44)	0.12 (−0.84–1.07)	0.07 (−0.93–1.07)	0.03 (−0.93–1.00)	**MB**	0.18 (−1.21–1.57)	1.02 (−0.38–2.43)
0.78 (−0.29–1.85)	0.64 (−0.06–1.34)	0.40 (−0.04–0.84)	0.35 (−0.22–0.92)	0.32 (−0.21–0.84)	0.28 (−0.62–1.18)	**BA**	0.00 (−1.42–1.42)
1.31 (0.18–2.44)	1.17 (0.53–1.81)	0.92 (0.52–1.33)	0.88 (0.45–1.31)	0.84 (0.50–1.19)	0.81 (−0.12–1.74)	0.53 (0.06–1.00)	**Control**

MB + BA, moxibustion with body acupuncture; EA + SA + BA, electroacupuncture with scalp acupuncture with body acupuncture; SA + BA, scalp acupuncture with body acupuncture; EA + SA, electroacupuncture with scalp acupuncture; MB, moxibustion; BA, body acupuncture. The estimates of mean difference of treatments in the columns vs. rows are presented in the lower diagonal elements, whereas those of the row treatments vs. column treatments are presented in the upper diagonal elements. The . symbol indicates results not reported in the studies.

**Figure 4:**
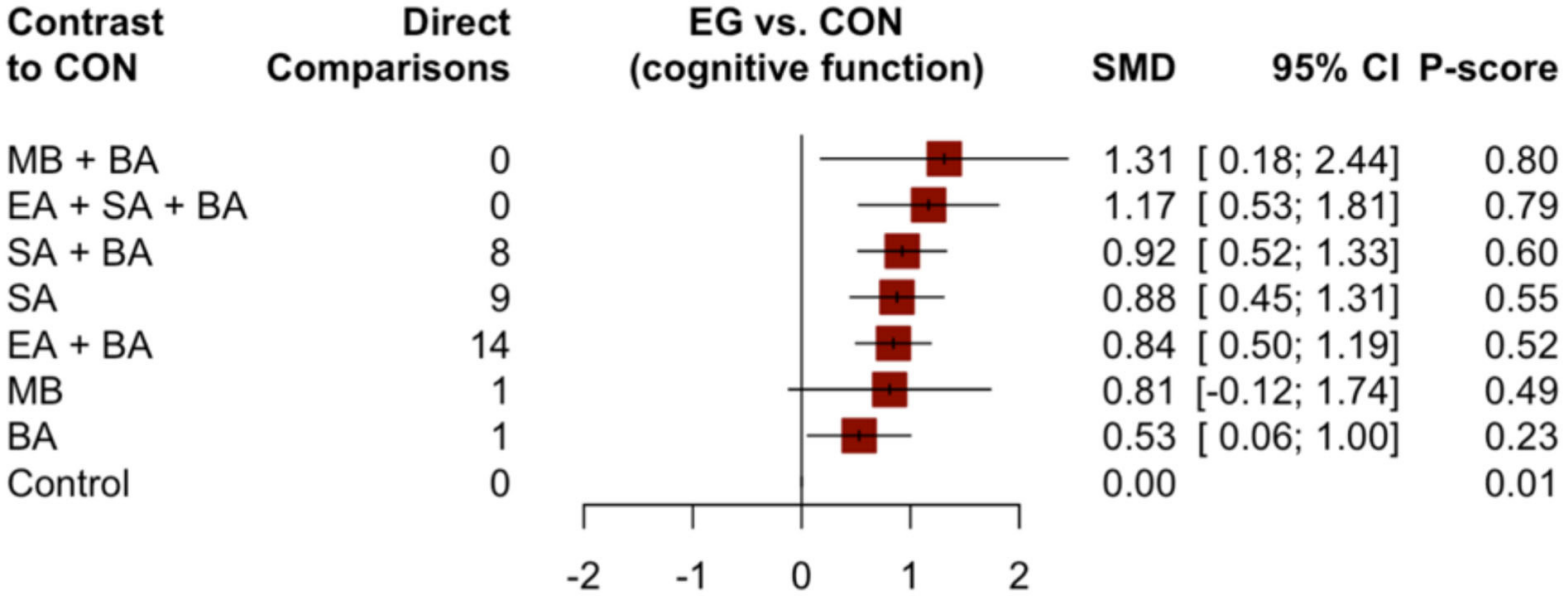
Forest plot compared with the control group (cognitive function).

Compared to simple SA and BA, the combined interventions EA + SA + BA (0.29, 95% CI: −0.46–1.04 and 0.64, 95% CI: −0.06–1.34, respectively) and SA + BA (0.05, 95% CI: −0.51–0.61 and 0.40, 95% CI: −0.04–0.84, respectively) showed better improvement in cognitive function. Compared to simple MB, the combined intervention MB + BA (0.50, 95% CI: −0.57–1.57) showed better improvement in cognitive function. SA may be more effective in relieving dementia symptoms than BA (0.35, 95% CI: −0.22–0.92). However, there were no appreciable differences between these acupuncture therapies in terms of improving cognitive function. However, acupuncture combined with EA or MB was better than SA or BA alone. Moreover, MB + BA showed the greatest possibility of becoming the most effective intervention for improving cognitive function (P-score = 0.80, Figure 4).

#### Inconsistency between direct and indirect comparisons of cognitive function scores

Inconsistency between direct and indirect comparisons was assessed using a node-splitting model, showing no inconsistencies among the studies (*P* > 0.05). Details of the results are presented in Table 3.

**Table 3:** Node-splitting analysis of inconsistency (cognitive function).

**Comparison**	**Direct RoM 95% CI**	**Indirect RoM 95% CI**	**Network RoM 95% CI**	***P*-value**
BA vs. EA + SA	−0.60 (−1.59–0.39)	−0.20 (−0.82–0.41)	−0.39 (−1.56–0.77)	0.554
BA vs. SA	−0.49 (−1.48–0.50)	−0.28 (−0.98–0.42)	−0.21 (−1.43–1.00)	0.983
BA vs. SA + BA	−0.23 (−0.76–0.31)	−0.74 (−1.51–0.03)	0.52 (−0.42–1.46)	0.171
EA + SA vs. EA + SA + BA	0.56 (−0.38–1.50)	−1.09 (−1.97–0.21)	1.65 (0.36–2.94)	1.000
EA + SA + BA vs. SA + BA	0.73 (0.03–1.44)	−0.92 (−2.00–0.16)	1.65 (0.36–2.94)	0.268
BA vs. MB	−0.18 (−1.57–1.21)	−0.35 (−1.54–0.83)	0.17 (−1.65–2.00)	0.853
EA + SA vs. Control	0.66 (0.29–1.04)	1.83 (0.95–2.70)	−1.16 (−2.11–0.21)	0.000
SA vs. Control	0.85 (0.38–1.31)	1.06 (−0.06–2.18)	−0.21 (−1.43–1.00)	0.732

RoM, ratio of mean.

#### Subgroup analysis

**Figure 5:**
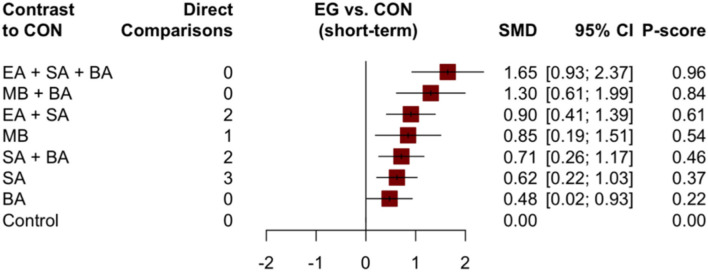
Forest plot compared with the control group of cognitive function (short-term).

An improvement in cognition scores in the short-term (1 ≤ × ≤ 30 days) was reported in 16 trials; 22 trials reported an improvement in cognition scores in the mid-term (30 < × ≤ 60 days), and eight trials reported an improvement in cognition scores in the long-term (× > 60 days). Data from different treatments were evaluated separately in accordance with different treatment durations in each subgroup. There were eight different interventions in the short-term. Paired meta-analysis showed that EA + SA + BA vs. BA (1.13, 95% CI: 0.43–1.84), SA + BA vs. EA + SA + BA (−0.93, 95% CI: −1.52– −0.35), control vs. EA + SA + BA (−1.53, 95% CI: −2.78– −0.27), and control vs. EA + SA (−0.88, 95% CI: −1.48– −0.28) were all statistically significantly different (Supplementary Appendix 7). In the NMA, the EA + SA + BA treatment showed the best improvement in cognitive function (0.96), followed by MB + BA (0.84), EA + SA (0.61), MB (0.54), SA + BA (0.46), SA (0.37), and BA (0.22). The details are illustrated in Figure 5. There were significant differences in the curative effects between EA + SA + BA and SA + BA (0.93, 95% CI: 0.37–1.49), SA (1.02, 95% CI: 0.24–1.80), and BA (1.17, 95% CI: 0.52–1.82, Supplementary Appendix 7).

**Figure 6:**
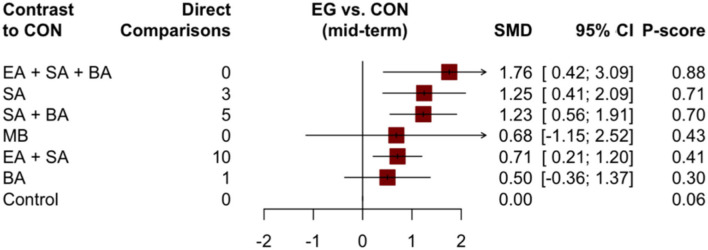
Forest plot compared with the control group of cognitive function (mid-term).

For the mid-term, there were no statistically significant differences between the groups in the paired meta-analysis (Supplementary Appendix 7). In the NMA, the highest probability of enhancing cognitive function was observed for EA + SA + BA, with a probability of 0.88, followed by SA and SA + BA with probabilities of 0.71 and 0.70, respectively (Figure 6, Supplementary Appendix 7). For the long-term, eight studies with five treatments (SA + BA, SA, EA + SA, control, and EA + SA + BA) were included. However, we failed to perform the corresponding network analysis because of the long-term network was disconnected.

Nine studies (Lai et al., 1998; Ling et al., 2007; Huang et al., 2012; Luo et al., 2015; Liu et al., 2016; Xie and Junming, 2016; Sheng and Cai, 2017; Feng, 2020; Ouyang, 2020) reported change scores using syndrome differentiation score of vascular dementia; however, failed to perform the corresponding network analysis due to the limited number of studies included.

### Outcome analysis of living ability

#### Pairwise meta-analysis

We performed a pairwise meta-analysis of living ability and the results are shown in Supplementary Appendix 3. The results of the pairwise meta-analysis showed that although each acupuncture therapy had slight advantages compared with the control group, there was no significant difference in any of the comparisons.

#### Network meta-analysis

**Figure 7:**
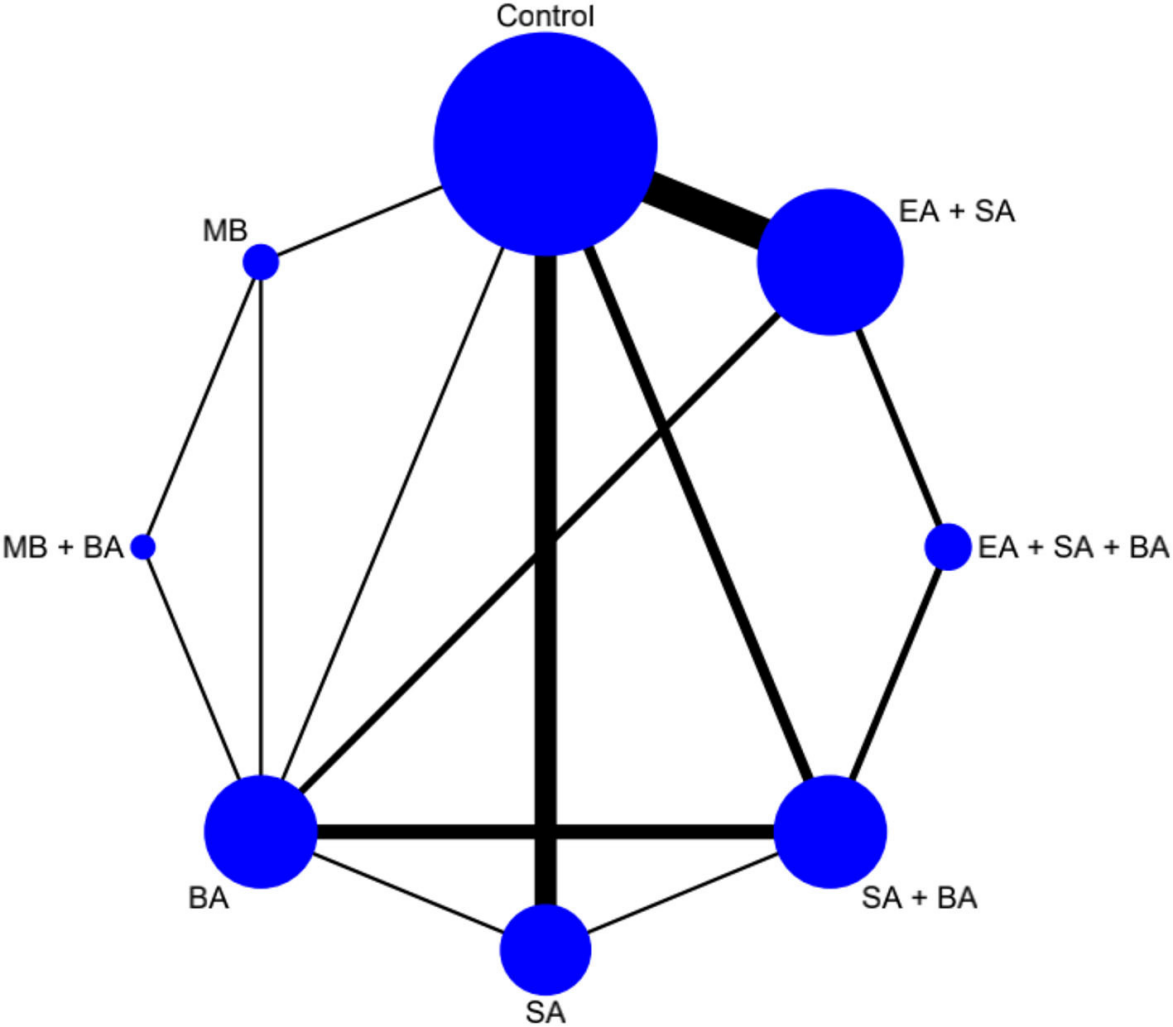
Network plot of living ability.

The network plot is shown in Figure 7. Eight interventions were involved: EA + SA + BA, SA + BA, EA + SA, SA, BA, MB + BA, MB, and control.

**Figure 8:**
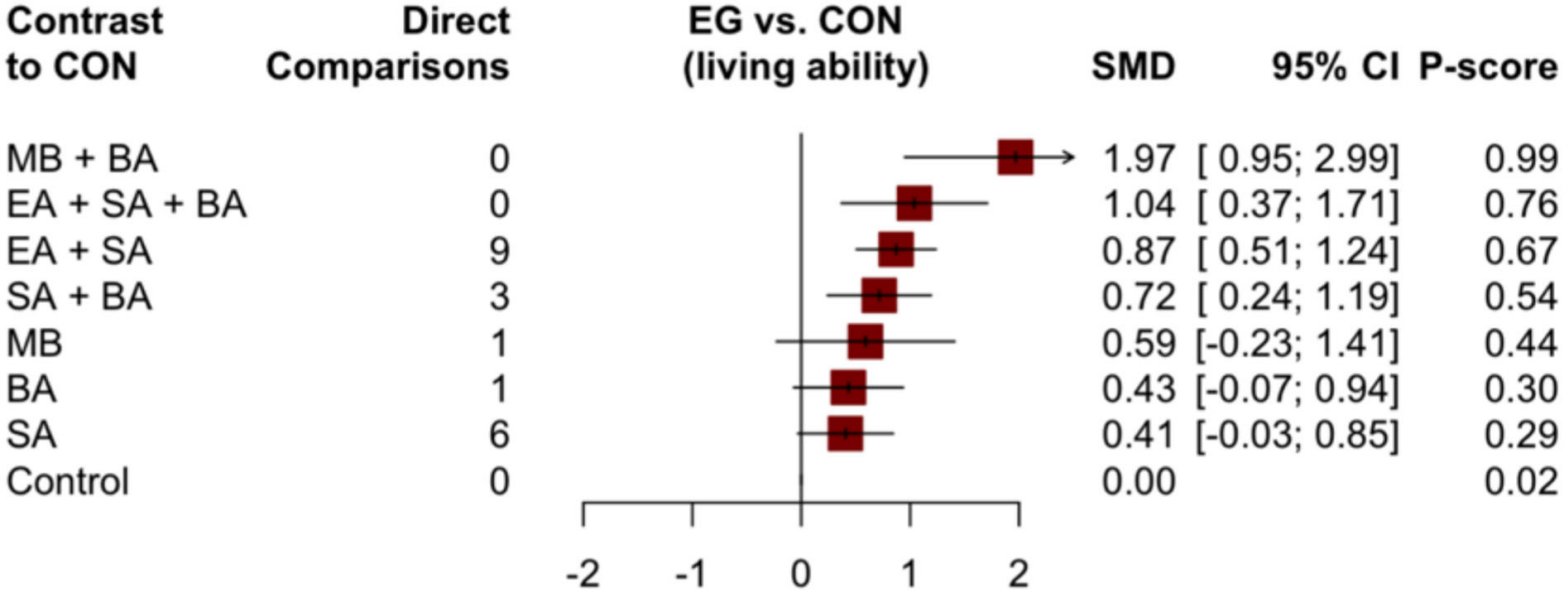
Forest plot compared with the control group (living ability).

Thirty studies with 2,486 patients in the main NMA reported changes in ADL scores using the ADLs and Barthel index. The results of the NMA of different treatments are shown in Table 4. Compared to the control group, MB + BA (1.97, 95% CI: 0.95–2.99), EA + SA + BA (1.04, 95% CI: 0.37–1.71), EA + SA (0.87, 95% CI: 0.51–1.24), and SA + BA (0.72, 95% CI: 0.24–1.19) showed better improvement in ADLs. However, other acupuncture treatments showed no significant difference compared to the control group. MA + BA may be more suitable for improving the activity of daily living compared to MB (1.38, 95% CI: 0.44–2.32) and BA (1.53, 95% CI: 0.59–2.47). Furthermore, combined acupuncture MB + BA (P-score = 0.99) and EA + SA + BA (P-score = 0.76, Figure 8) are likely to be the best intervention to improve the ADLs of patients with VaD.

**Table 4:** Results of network meta-analysis for all possible treatment effects (living ability).

**MB + BA**	.	.	.	1.42 (0.21–2.62)	1.50 (−0.29–2.70)	.	.
0.93 (−0.24– 2.10)	**EA + SA + BA**	0.13 (−0.93–0.68)	0.66 (−0.20–1.52)	.	.	.	.
1.10 (0.05–2.14)	0.17 (−0.47–0.80)	**EA + SA**	.	.	0.21 (−0.64–1.07)	.	0.85 (0.44–1.25)
1.25 (0.21–2.29)	0.32 (−0.33–0.97)	0.16 (−0.38–0.69)	**SA + BA**	.	0.21 (−0.46–0.88	−0.17 (−1.38–1.04)	1.16 (0.47–1.85)
1.38 (0.44–2.32)	0.45 (−0.56–1.46)	0.28 (−0.58–1.14)	0.13 (−0.75–1.00)	**MB**	0.12 (−1.06–1.30)	.	0.67 (−0.55–1.88)
1.53 (0.59–2.47)	0.61 (−0.13–1.34)	0.44 (−0.09–0.97)	0.28 (−0.23–0.79)	0.16 (−0.63–0.94)	**BA**	−0.20 (−1.43–1.02)	−0.12 (−1.36–1.11)
1.56 (0.49–2.64)	0.63 (−0.14–1.40)	0.47 (−0.09–1.02)	0.31 (−0.27–0.89)	0.18 (−0.71–1.08)	0.03 (−0.57–0.62)	**SA**	0.29 (−0.20–0.78)
1.97 (0.95–2.99)	1.04 (0.37–1.71)	0.87 (0.51–1.24)	0.72 (0.24–1.19)	0.59 (−0.23–1.41)	0.43 (−0.07–0.94)	0.41 (−0.03–0.85)	**Control**

MB + BA, moxibustion with body acupuncture; EA + SA + BA, electroacupuncture with scalp acupuncture with body acupuncture; SA + BA, scalp acupuncture with body acupuncture; EA + SA, electroacupuncture with scalp acupuncture; MB, moxibustion; BA, body acupuncture. The estimates of mean difference of treatments in the columns vs. rows are presented in the lower diagonal elements, whereas those of the row treatments vs. column treatments are presented in the upper diagonal elements. The . symbol indicates results not reported in the studies.

#### Inconsistency between direct and indirect comparisons of life function scores

Inconsistency between direct and indirect comparisons was assessed using a node-splitting model, showing no inconsistencies among the studies (*P* > 0.05). Details of the results are presented in Table 5.

**Table 5:** Node-splitting analysis of inconsistency (living ability).

**Comparison**	**Direct RoM 95% CI**	**Indirect RoM 95% CI**	**Network RoM 95% CI**	***P*-value**
BA vs. EA + SA	−0.21 (−1.07–0.64)	−0.58 (−1.26–0.10)	−0.37 (−0.72–1.46)	0.960
BA vs. SA	−0.20 (−1.43–1.02)	−0.10 (−0.58–0.78)	−0.30 (−1.70–1.10)	0.621
EA + SA vs. EA + SA + BA	0.13 (−0.68–0.93)	−0.67 (−1.72–0.38)	0.80 (−0.52–2.12)	1.000
EA + SA + BA vs. SA + BA	0.66 (−0.20–1.52)	−0.14 (−1.14 to −0.86)	0.80 (−0.52–2.12)	0.236
EA + SA vs. control	0.85 (0.44–1.25)	0.98 (0.12–1.84)	−0.13 (−1.08–0.81)	0.000
SA vs. control	0.29 (−0.20–0.78)	0.86 (−0.11–1.84)	−0.57 (−1.67–0.52)	0.569

RoM, ratio of mean.

### Adverse events

Forty participants from eight studies (Zhang et al., 2008; Huang et al., 2012; Shi et al., 2014; Wang, 2014; Wang et al., 2018; Feng, 2020; Ouyang, 2020; Yao, 2020) reported the presence of adverse events. The main adverse effects reported in the acupuncture group were “discomfort at the acupuncture site” (16 patients), “skin bruising at acupoints” (15 patients), “fainting during acupuncture treatment” (seven patients), and “bleeding at acupuncture points” (two patients). These symptoms are mild and persist for a short period of time.

### Confidence in evidence

The confidence ratings for these comparisons with CINeMA (Supplementary material) were mostly low to moderate confidence. This was mainly because of within-study bias, lack of precision, and/ or heterogeneity. Poor reporting of the randomization and blinding procedures explained the concerns of within-study bias. The relatively small number of studies with direct evidence included in all the comparisons explains the imprecision. The observed heterogeneity is likely because of the small number of trials in some comparisons, whereas some of the RCTs focused on different subgroups of VaD.

## Discussion

To the best of our knowledge, this study is the first NMA to explore the efficacy of different acupuncture treatments for VaD. The MMSE scale (Cui et al., 2019) is currently the most widely used neuropsychological scale for clinical cognitive function screening, which can comprehensively reflect the cognitive state and cognitive characteristics of patients. Cognitive impairment is a core symptom of VaD; therefore, we chose to assess changes in cognitive impairment as the primary efficiency outcome of this review. In addition, the decline in the ability to perform ADLs is also one of the main symptoms of VaD; thus, we choose the ADLs as secondary efficacy outcome indicators. We used direct and indirect evidence to evaluate the relative effects of different acupuncture therapies on cognitive function and the ability to perform ADLs in patients with VaD. Based on the currently available data, our NMA suggests that combined acupuncture interventions (EA + SA + BA and MB + BA) show better efficacy in improving cognitive function and the ability to perform ADLs compared with MB, SA, and BA alone. SA was more effective than BA in improving cognitive function. Using a subgroup analysis of cognitive function, we found that EA + SA + BA achieved the best effect among the therapies analyzed for both the short- and mid-terms. However, for the short-term, EA + SA was more effective than SA + BA, whereas the opposite was true for the mid-term. This may be why there is a certain degree of heterogeneity in our review. Furthermore, the efficacy of EA in improving the ADLs was better than that of non-EA treatments.

Based on the analysis of adverse reactions from all included studies, all included acupuncture treatments were relatively safe for patients with VaD. Although some cases of adverse reactions have been reported, these adverse reactions are mild, and there is no direct association between adverse reactions and interventions. Therefore, these cases were justified.

Numerous studies have reported that VaD may be caused by hypoperfusion (Ruitenberg et al., 2005) and hypoxia (Fernando et al., 2006) after cerebral ischemia, resulting in alterations in blood–brain barrier permeability (Candelario-Jalil et al., 2011; Wardlaw et al., 2013), oxidative stress, and inflammation (Gallacher et al., 2010; Rouhl et al., 2012), which in turn can lead to white matter damage (Iadecola et al., 2019). The exact mechanism by which EA + SA + BA and MB + BA show better therapeutic efficiency in patients with VaD is still not fully understood. However, studies have shown that EA can enhance the secretion of brain-derived neurotrophic factors to exert neuroprotective effects after cerebral ischemia (Tao et al., 2016). EA can reduce oxidative stress and anti-apoptotic cell death by increasing superoxide dismutase activity in the brain (Wang et al., 2011). MB can inhibit apoptosis and oxidative stress and improve vascular endothelial growth factor inflammation (Lai et al., 2022). MB may improve cognitive function in VaD patients by inhibiting hippocampal neuronal apoptosis (Yang et al., 2021). SA can improve blood supply to the brain and enhance neuronal metabolism (Chen et al., 2020). EA + SA can improve the learning and memory ability of VaD rats by down-regulating inflammatory factors, reducing neuronal apoptosis, and improving synaptic plasticity (Ma et al., 2022). SA + BA can improve cognitive function by inhibiting brain oxidative stress and inflammation, alleviating neuronal death in VaD rats (Du et al., 2018). Therefore, we speculate that the reason why EA + SA + BA and MB + BA are superior to other acupuncture treatments may be that the combination therapy brings together the advantages of individual acupuncture treatments. However, to determine whether these findings are attributed to simple additive effects or due to the interaction of multiple signaling pathways *in vivo* requires further research.

### Limitations

This study had the following limitations: (i) the included RCTs were mainly conducted in China, (ii) Reliability of results may be affected by incomplete reporting of test details, (iii) testing indexes other than the scale could not be included because of the limited number of studies, and (iv) due to the limited number of studies retrieved, we could not extend our review more beneficially, such as whether the analysis results varied by patient sex (male vs. female), or whether effect size corresponds to the atherosclerotic cardiovascular disease (ASCVD) burden.

The lack of long-term follow-up studies makes it hard to conclude substantial implications of the study. The disease cycle in patients with VaD usually has longer chronic progression. More evidence is required to confirm whether acupuncture has long-term efficacy in addition to short-term improvement in cognitive function.

## Conclusion

Combined acupuncture therapy may be an effective and safe intervention for patients with VaD, and MB + BA and EA + SA + BA emerged as the most effective interventions in this study. EA + SA + BA shows the best efficacy when the course of acupuncture is short or moderate. However, because the analysis in this study was based on low-to-moderate evidence, there is no strong supporting evidence in this regard. High-quality, large-scale, long-term studies should be performed in the future to investigate the efficacy and safety of acupuncture in VaD.

## Data availability statement

The datasets presented in this study can be found in online repositories. The names of the repository/repositories and accession number(s) can be found in the article/Supplementary material.

## Author contributions

JW: research design and writing. YC, HP, and HL: guidance of the relevant methodology. JW, SC, and ZZ: study selection. JW, QH, and JY: data abstraction. WW, SC, and QH: quality assessment. JW and SC: data statistics and analysis. All authors examined the results of this study and approved the final submission.
