# Supplementary material for: A network meta-analysis on the improvement of cognition in patients with vascular dementia by different acupuncture therapies
Source: Front Neurosci. 2022 Dec 14;16:1053283. doi: 10.3389/fnins.2022.1053283 (PMC9797048; doi:10.3389/fnins.2022.1053283)
Supplement: Supplementary file 1 [file Data_Sheet_1.PDF]

Supplementary Material

Appendix 1 Search strategy 2

Appendix 2 Risk of Bias Assessment 4

Appendix 3 Pairwise Meta-analysis 5

Appendix 4 Evaluation of heterogeneity and inconsistency 7

Appendix 5 Details of splitting results 8

Appendix 6 Summary of network pooled results and confidence in evidence 15

Appendix 7 Subgroup analysis 18

Appendix 1 Search strategy

Table 1. PubMed search terms

#1 “Acupuncture” [Mesh], #2 “Acupuncture Therapy” [Mesh], #3 “Acupuncture” [Title/Abstract], #4 “Acupuncture Therapy” [Title/Abstract], #5 “Acupuncture Treatment” [Title/Abstract], #6 “Acupoint* [Title/Abstract], #7 “Acupuncture*” [Title/Abstract], #8 “Needling” [Title/Abstract], #9 “Electroacupuncture” [Title/Abstract], #10 “Electro-acupuncture” [Title/Abstract], #11 “Electrostimulation” [Title/Abstract], #12 “Moxibustion” [Title/Abstract], #13 “Scalp Acupuncture” [Title/Abstract], #14 “Auriculoacupuncture” [Title/Abstract], #15 OR #1-14
#16 “Vascular Dementia” [Mesh], #17 “Vascular Dementia” [Title/Abstract], #18 “Subcortical Vascular Dementia” [Title/Abstract], #19 “Infarct dementia” [Title/Abstract], #20 “Post-stroke dementia” [Title/Abstract], #21 “Vascular cognitive impairment“ [Title/Abstract], #22 OR #16-21
#23 “Randomized Controlled Trial” [Publication Type] OR “Randomized Controlled Trials as Topic” [Mesh] OR “Randomized Controlled Trials” [Title/Abstract] OR “Single-Blind Method” [Mesh] OR “double-blind Method” [Mesh], #24 “Controlled Clinical Trial” [Publication Type] OR “Controlled Clinical Trials as Topic” [Mesh] OR “Controlled Clinical Trial*” [Title/Abstract] OR “Controlled Trial*” [Title/Abstract] OR “Controlled stud*” [Title/Abstract], #25 OR #26-27
#28 #15 AND #22 AND #25 AND 1990:2022 [Publication Type]

Table 2. Cochrane search terms

#1 Mesh descriptor: [Vascular Dementia] explode all trees, #2 Vascular Dementia: Title, Abstract, Key Words, #3 Subcortical Vascular Dementia: Title, Abstract, Key Words, #4 Infarct dementia: Title, Abstract, Key Words, #5 Post-stroke dementia: Title, Abstract, Key Words, #6 Vascular cognitive impairment: Title, Abstract, Key Words, #7 OR #1-6
#8 Mesh descriptor: [Acupuncture] explode all trees, #9 Mesh descriptor: [Acupuncture Therapy] explode all trees, #10 Acupuncture: Title, Abstract, Key Words, #11 Acupuncture Therapy: Title, Abstract, Key Words, #12 Acupuncture Treatment: Title, Abstract, Key Words, #13: Acupoint*: Title, Abstract, Key Words, #14: Needling: Title, Abstract, Key Words, #15: Electroacupuncture: Title, Abstract, Key Words, #16: Electro-acupuncture: Title, Abstract, Key Words, #17: Electrostimulation: Title, Abstract, Key Words, #18: Moxibustion: Title, Abstract, Key Words, #19: Scalp Acupuncture: Title, Abstract, Key Words, #20: OR #8-19
#21 Randomized Controlled Trials: Title, Abstract, Key Words, #22 Controlled Clinical Trials: Title, Abstract, Key Words, #23: OR #21-22
#24: #7 AND #20 AND #23

Table 3. Embase search terms (and MEDLINE, Web of Science)

#1 ‘vascular dementia’: ab, ti, #2 ‘subcortical vascular dementia’: ab, ti, #3 ‘infarct dementia’: ab, ti, #4 ‘post-stroke dementia’: ab, ti, #5 ‘vascular cognitive impairment’: ab, ti, #6 OR #1-5

#7 ‘acupuncture’: ab, ti, #8 ‘acupuncture therapy’: ab, ti, #9 ‘acupuncture treatment’: ab, ti, #10 ‘acupoint*’: ab,ti, #11 ‘needling’: ab, ti, #12 ‘electroacupuncture’ : ab, ti, #13 ‘electro-acupuncture’ : ab, ti, #14 ‘electrostimulation’ : ab, ti, #15 ‘scalp acupuncture’ : ab, ti, #16 ‘moxibustion’ : ab, ti, #17 OR #7-16

#18 ‘randomized controlled trials’ : ab, ti, #19 ‘controlled clinical trials’ : ab, ti, #20 #18 OR #19

#21 #6 AND #17 AND #20

CNKI search terms: The Chinese version available on request from reviewers or readers.

Appendix 2 Risk of Bias Assessment

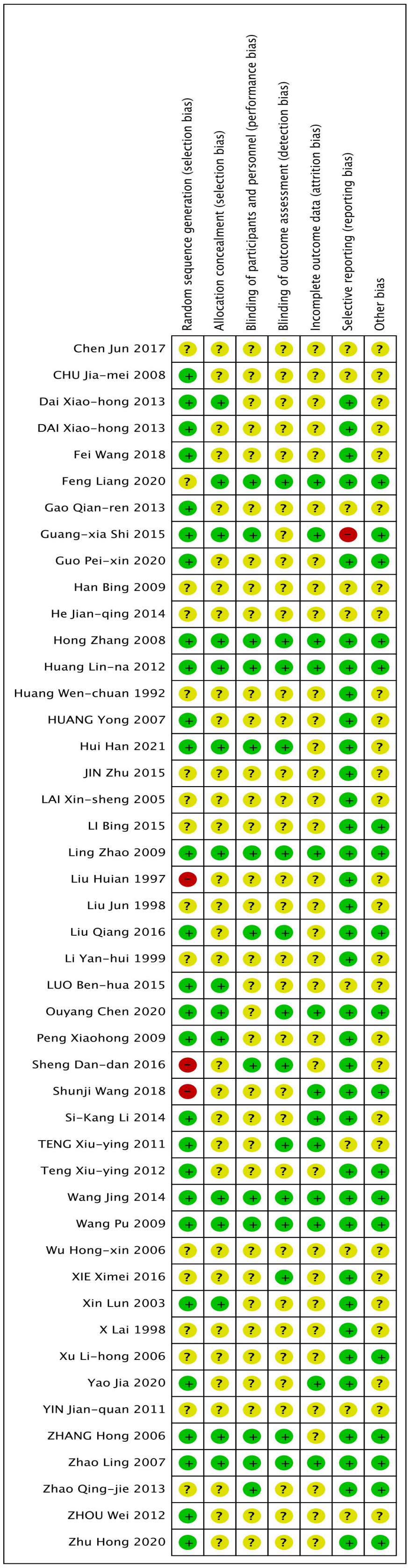

Appendix 3 Pairwise meta-analysis

Figure 1 Pairwise meta-analysis of cognitive function

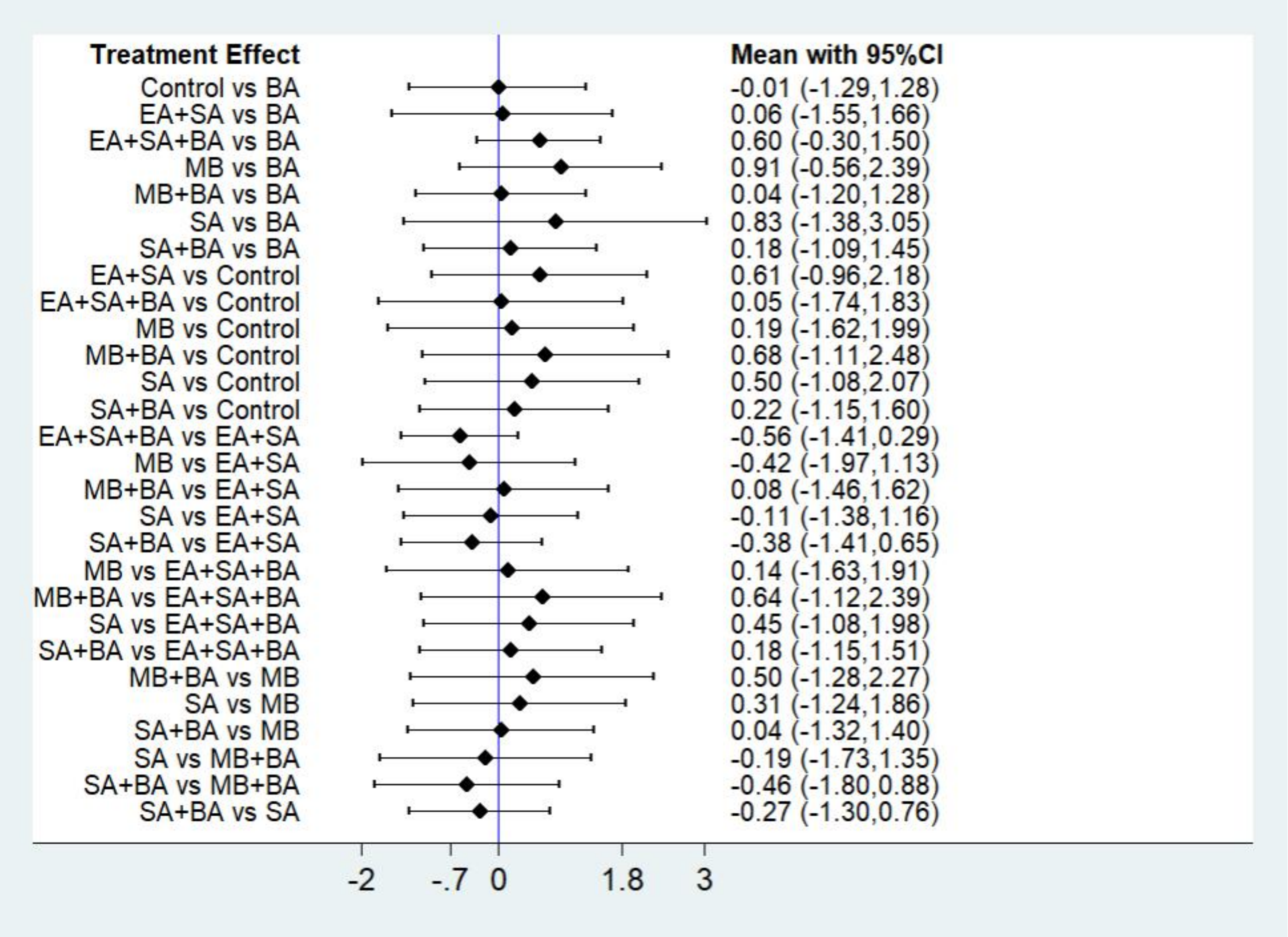

Figure 2 Pair-wise meta-analysis of living ability

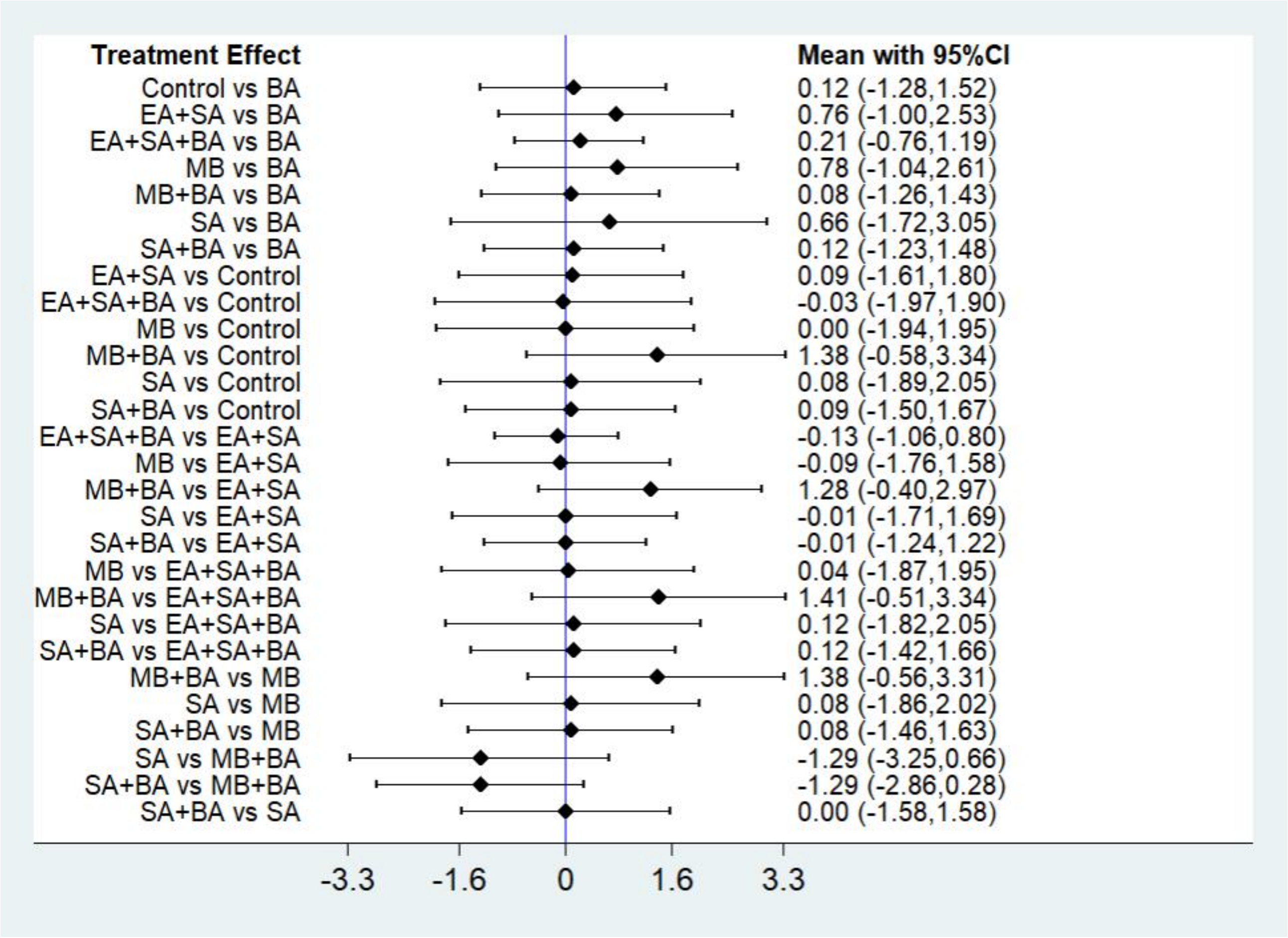

Appendix 4 Evaluation of heterogeneity and inconsistency

Table 1. Evaluation of heterogeneity and inconsistency (cognitive function and ability of daily life)

**Outcomes**	**Number of studies**	**Heterogeneity**					**Inconsistency**			
		**t^2**	**Q**	**df**	**P**	**I^2**	**Q**	**df**	**t^2**	**P-value**
**Cognitive function**	47	0.438	349.48	47	<0.0001	86.6%	13.11	7	0.345	0.070
**Ability of daily life**	30	0.312	164.13	28	<0.0001	82.9%	4.6	7	0.330	0.709

Table 2. Evaluation of heterogeneity and inconsistency (subgroup analysis of cognitive function)

Outcomes	Number of studies	heterogeneity					Inconsistency			
		t^2	Q	df	P	I2	Q	df	t^2	P-value
Short-term	17	0.089	27.060	14	0.019	48.3%	1.45	3	0.105	0.695
Mid-term	22	0.613	205.65	19	<0.0001	90.8%	1.76	3	0.660	0.623

Appendix 5 Details of splitting results

Table 1. Details of splitting results (cognitive function)

comparison	k	prop	NMA		Direct		Indirect		Diff		p	z
			TE	se TE	TE	se TE	TE	se TE	TE	se TE		
BA vs control	1	0.112242297	0.520271364	0.242642711	0	0.724250789	0.586051084	0.257525373	–0.586051084	0.768673223	0.445809959	–0.762419019
BA vs EA + SA	2	0.283709318	–0.316331085	0.268719995	–0.599064597	0.504502338	–0.20434564	0.317508482	–0.394718957	0.596099191	0.507862326	–0.662169926
BA vs EA + SA + BA	0	0	–0.595806112	0.336111274	NA	NA	–0.595806112	0.336111274	NA	NA	NA	NA
BA vs MB	1	0.419094204	–0.283732299	0.459835441	–0.17997065	0.710307848	–0.358591091	0.603322589	0.178620441	0.931952459	0.848006495	0.191662611
BA vs MB + BA	1	0.606971298	–0.783597617	0.547238725	–0.67683822	0.702413331	–0.948470793	0.872900393	0.271632573	1.120419378	0.808440558	0.242438303
BA vs SA	2	0.33430413	–0.354594271	0.292648552	–0.489386093	0.506145651	–0.286903506	0.358681063	–0.202482587	0.620351131	0.744121765	–0.326399964
BA vs SA+BA	8	0.679692882	–0.39121379	0.225360916	–0.22631754	0.27335199	–0.74112424	0.398194544	0.514806695	0.482990895	0.286481298	1.065872463

			8		9		4					
EA + SA vs control	14	0.836846884	0.83660245	0.17433963	0.664295848	0.190578156	1.720399499	0.431616893	−1.056103651	0.471819008	0.025197194	−2.238366055
EA + SA + BA vs control	1	0.19112737	1.116077477	0.29709496	0.89335611	0.679569444	1.168703994	0.330335474	−0.275347884	0.755603173	0.71555332	−0.364408056
MB vs control	1	0.435967356	0.804003664	0.473230525	1.020796	0.716713797	0.636434672	0.630116179	0.384361328	0.954319163	0.687124989	0.40275973
MB + BA vs control	0	0	1.303868982	0.578915679	NA	NA	1.303868982	0.578915679	NA	NA	NA	NA
SA vs control	9	0.854229494	0.874865636	0.21890702	0.845349646	0.236849465	1.047832233	0.573356657	−0.202482587	0.620351131	0.744121765	−0.326399964
SA + BA vs control	7	0.606990003	0.911485162	0.212395641	1.381368201	0.27261819	0.185767474	0.338800311	1.195600727	0.434863574	0.005971	2.749369684
EA + SA vs EA + SA + BA	2	0.405066429	−0.279475027	0.305865327	0.55966282	0.480581581	−0.850810361	0.396548224	1.410473181	0.623064323	0.023588377	2.263768169
EA + SA vs MB	0	0	0.032598786	0.493964593	NA	NA	0.032598786	0.493964593	NA	NA	NA	NA
EA + SA vs MB + BA	0	0	−0.467266532	0.593160847	NA	NA	−0.467266532	0.593160847	NA	NA	NA	NA
EA + SA vs SA	0	0	−0.038263186	0.274317472	NA	NA	−0.038263186	0.274317472	NA	NA	NA	NA

EA + SA vs SA + BA	0	0	−0.074882712	0.251532261	NA	NA	−0.074882712	0.251532261	NA	NA	NA	NA
EA + SA + BA vs MB	0	0	0.312073813	0.539907913	NA	NA	0.312073813	0.539907913	NA	NA	NA	NA
EA + SA + BA vs MB + BA	0	0	−0.187791505	0.62926286	NA	NA	−0.187791505	0.62926286	NA	NA	NA	NA
EA + SA + BA vs SA	0	0	0.241211841	0.360620903	NA	NA	0.241211841	0.360620903	NA	NA	NA	NA
EA + SA + BA vs SA + BA	4	0.626317819	0.204592315	0.283707184	0.733493351	0.358486619	−0.681883306	0.46410809	1.415376656	0.586437529	0.015799412	2.413516509
MB vs MB + BA	1	0.607440518	−0.499865318	0.54712332	−0.60649726	0.701993915	−0.334864687	0.873237726	−0.271632573	1.120419378	0.808440558	−0.242438303
MB vs SA	0	0	−0.070861972	0.508965526	NA	NA	−0.070861972	0.508965526	NA	NA	NA	NA
MB vs SA + BA	0	0	−0.107481498	0.486633636	NA	NA	−0.107481498	0.486633636	NA	NA	NA	NA
MB + BA vs SA	0	0	0.429003346	0.605044697	NA	NA	0.429003346	0.605044697	NA	NA	NA	NA
MB + BA vs SA	0	0	0.39238382	0.58098040	NA	NA	0.39238382	0.58098040	NA	NA	NA	NA

+BA				7				7				
SA vs SA + BA	0	0	−0.036619527	0.288921195	NA	NA	−0.036619527	0.288921195	NA	NA	NA	NA

Table 2. Details of splitting results (ability of daily life)

comparison	k	prop	NMA		Direct		Indirect		Diff		p	z
			TE	se TE	TE	se TE	TE	se TE	TE	se TE		
BA vs control	1	0.164801196	0.433881253	0.256506503	−0.12358134	0.631856127	0.543879617	0.280674817	−0.667460957	0.691390279	0.334349728	−0.96538956
BA vs EA+SA	2	0.387126636	−0.437682816	0.271453826	−0.211762843	0.436284154	−0.580387075	0.346745308	0.368624232	0.557293614	0.508321042	0.661454255
BA vs EA + SA + BA	0	0	−0.605245465	0.377010349	NA	NA	−0.605245465	0.377010349	NA	NA	NA	NA
BA vs MB	1	0.43854604	−0.156850211	0.39999779	−0.12106956	0.604018159	−0.184798118	0.533826977	0.063728558	0.806107423	0.936987164	0.079057153
BA vs MB + BA	1	0.604656073	−1.53453945	0.479486334	− 1.496188	0.616626271	−1.59319582	0.762585771	0.097007823	0.98069619	0.92120393	0.098917304

			4				3					
BA vs SA	1	0.238337952	0.027647088	0.304710233	–0.20225862	0.624152103	0.099588778	0.349145044	–0.301847398	0.715169987	0.672978413	–0.422063851
BA vs SA + BA	4	0.575611735	–0.28263417	0.260569842	–0.21178776	0.343446686	–0.378725481	0.39998386	0.166937721	0.527202726	0.751510664	0.316648061
EA + SA vs control	9	0.818625173	0.871564069	0.186627237	0.847166919	0.206268295	0.981679247	0.438214274	–0.134512328	0.484332902	0.781221926	–0.277727008
EA + SA + BA vs control	0	0	1.039126718	0.342433216	NA	NA	1.039126718	0.342433216	NA	NA	NA	NA
MB vs control	1	0.455232133	0.590731465	0.417445853	0.66688379	0.6187051	0.527095212	0.565580477	0.139788578	0.838258479	0.867558335	0.166760709
MB + BA vs control	0	0	1.968420707	0.519507472	NA	NA	1.968420707	0.519507472	NA	NA	NA	NA
SA vs control	6	0.797459893	0.406234165	0.223969142	0.290030318	0.250803598	0.863762846	0.497659925	–0.573732529	0.557286143	0.303239349	–1.029511563
SA + BA vs control	3	0.477731269	0.716515423	0.242958726	1.163577907	0.351512415	0.307577045	0.336190541	0.856000862	0.4864001	0.07842988	1.759869831

EA + SA vs EA + SA + BA	2	0.629629162	−0.167562649	0.325015344	0.12777311	0.409601414	−0.669632433	0.534054369	0.797405543	0.673043377	0.236106081	1.184775856
EA +SA vs MB	0	0	0.280832605	0.438978301	NA	NA	0.280832605	0.438978301	NA	NA	NA	NA
EA + SA vs MB + BA	0	0	−1.096856638	0.532037741	NA	NA	−1.096856638	0.532037741	NA	NA	NA	NA
EA + SA vs SA	0	0	0.465329904	0.281487155	NA	NA	0.465329904	0.281487155	NA	NA	NA	NA
EA + SA vs SA + BA	0	0	0.155048646	0.271978842	NA	NA	0.155048646	0.271978842	NA	NA	NA	NA
EA + SA + BA vs MB	0	0	0.448395254	0.51635915	NA	NA	0.448395254	0.51635915	NA	NA	NA	NA
EA + SA + BA vs MB + BA	0	0	−0.929293989	0.595194519	NA	NA	−0.929293989	0.595194519	NA	NA	NA	NA
EA + SA + BA vs SA	0	0	0.632892553	0.392759094	NA	NA	0.632892553	0.392759094	NA	NA	NA	NA
EA + SA + BA vs SA + BA	2	0.57592089	0.322611295	0.332619656	0.660774328	0.438294978	−0.136631215	0.510768929	0.797405543	0.673043377	0.236106081	1.184775856

MB vs MB + BA	1	0.606136327	–1.377689242	0.479173297	-1.4158971	0.615470797	–1.318889277	0.763518641	–0.097007823	0.98069619	0.92120393	–0.098917304
MB vs SA	0	0	0.184497299	0.45865426	NA	NA	0.184497299	0.45865426	NA	NA	NA	NA
MB vs SA + BA	0	0	–0.125783959	0.44570818	NA	NA	–0.125783959	0.44570818	NA	NA	NA	NA
MB + BA vs SA	0	0	1.562186542	0.549066677	NA	NA	1.562186542	0.549066677	NA	NA	NA	NA
MB + BA vs SA + BA	0	0	1.251905284	0.532124816	NA	NA	1.251905284	0.532124816	NA	NA	NA	NA
SA vs SA + BA	1	0.23018552	–0.310281258	0.295885236	0.16788124	0.616714693	–0.453258671	0.337233149	0.621139911	0.7028963	0.376865516	0.883686414

Appendix 6 Summary of network pooled results and confidence in evidence

	Cognitive function		Ability of daily life	
	NMA estimate (95% CI)	CINeMA confidence rating	NMA estimate (95% CI)	CINeMA confidence rating
BA vs EA + SA	-1.11 (-2.87, 0.64)	Low	-0.68 (-3.85, 2.50)	Low
BA vs MB	-1.25 (-4.07, 1.58)	Low	-0.79 (-4.97, 3.40)	Low
BA vs MB + BA	-4.29 (-7.92, -0.66)	Low	-11.84 (-16.83, -6.85)	Moderate
BA vs SA	-1.42 (-3.33,0.48)	Low	-0.39 (-3.28, 4.06)	Low
BA vs SA + BA	-1.66 (-3.21, -0.11)	Low	-3.02 (-5.78, -0.26)	Low
BA vs Control	1.58 (-0.04, 3.19)	Low	3.87 (-0.83, 6.90)	Moderate
EA + SA vs EA + SA + BA	-1.31 (-3.32, 0.69)	Low	-2.09 (-5.32, 1.13)	Very low
Control vs EA + SA	-2.69 (-3.79, -1.59)	Moderate	-4.55 (-6.47, -2.63)	Moderate
EA + SA + BA vs SA + BA	0.77 (-1.10, 2.63)	Low	-0.25 (-3.65, 3.16)	Low
MB vs MB + BA	-3.04 (-6.68, 0.59)	Very low	-11.05 (-16.03, -6.08)	Moderate
Control vs MB	-2.83 (-5.77, 0.12)	Very low	-4.65 (-9.05, -0.26)	Low
Control vs SA	-3.00 (-4.37, -	Moderate	-3.48 (-6.18, -	Moderate

	1.63)		0.78)	
Control vs SA + BA	-3.24 (-4.54, -1.93)	Moderate	-6.89 (-9.73, -4.04)	Low
BA vs EA + SA + BA	-2.43 (-4.71, -0.14)	Moderate	-2.78 (-6.69, 1.15)	Very low
EA + SA vs MB	-0.14 (-3.20, 2.93)	Low	-0.11 (-4.72, 4.50)	Very low
EA + SA vs MB + BA	-3.18 (-7.10, 0.75)	Low	-11.16 (-16.80, -5.52)	Moderate
EA + SA vs SA	-0.31 (-2.03, 1.41)	Low	1.07 (-2.16, 4.30)	Low
EA + SA vs SA + BA	-0.55 (-3.00, 1.00)	Low	-2.34 (-5.38, 0.70)	Very low
EA + SA + BA vs MB	1.18 (-2.26, 4.61)	Low	1.98 (-3.29, 7.26)	Low
EA + SA + BA vs MB + BA	-1.87 (-6.07, 2.34)	Low	-9.07 (-15.21, -2.93)	Moderate
EA + SA + BA vs SA	1.00 (-1.38, 3.39)	Low	3.16 (-1.08, 7.40)	Low
Control vs EA + SA + BA	-4.00 (-6.02, -2.00)	Moderate	-6.64 (-10.10, -3.17)	Low
MB vs SA	-0.18 (-3.33,3.00)	Low	1.18 (-3.80, 6.15)	Low
MB vs SA + BA	-0.41 (-3.44, 2.61)	Low	-2.23 (-6.88, 2.42)	Low
MB + BA vs SA	2.87 (-1.13, 6.87)	Low	12.23 (6.29, 18.16)	Low
MB + BA vs SA + BA	2.63 (-1.24, 6.50)	Low	8.82 (3.28, 14.36)	Low
Control vs MB +	-5.87 (-9.72, -	Low	-15,71 (-21,22, -	Very low

BA	2.02)		10.20)	
SA vs SA + BA	-0.24 (-2.04, 1.57)	Low	-3.41 (-7.03, 0.21)	Low

Appendix 7. Subgroup analysis

Figure 1. Pair-wise meta-analysis of cognitive function (short-term)

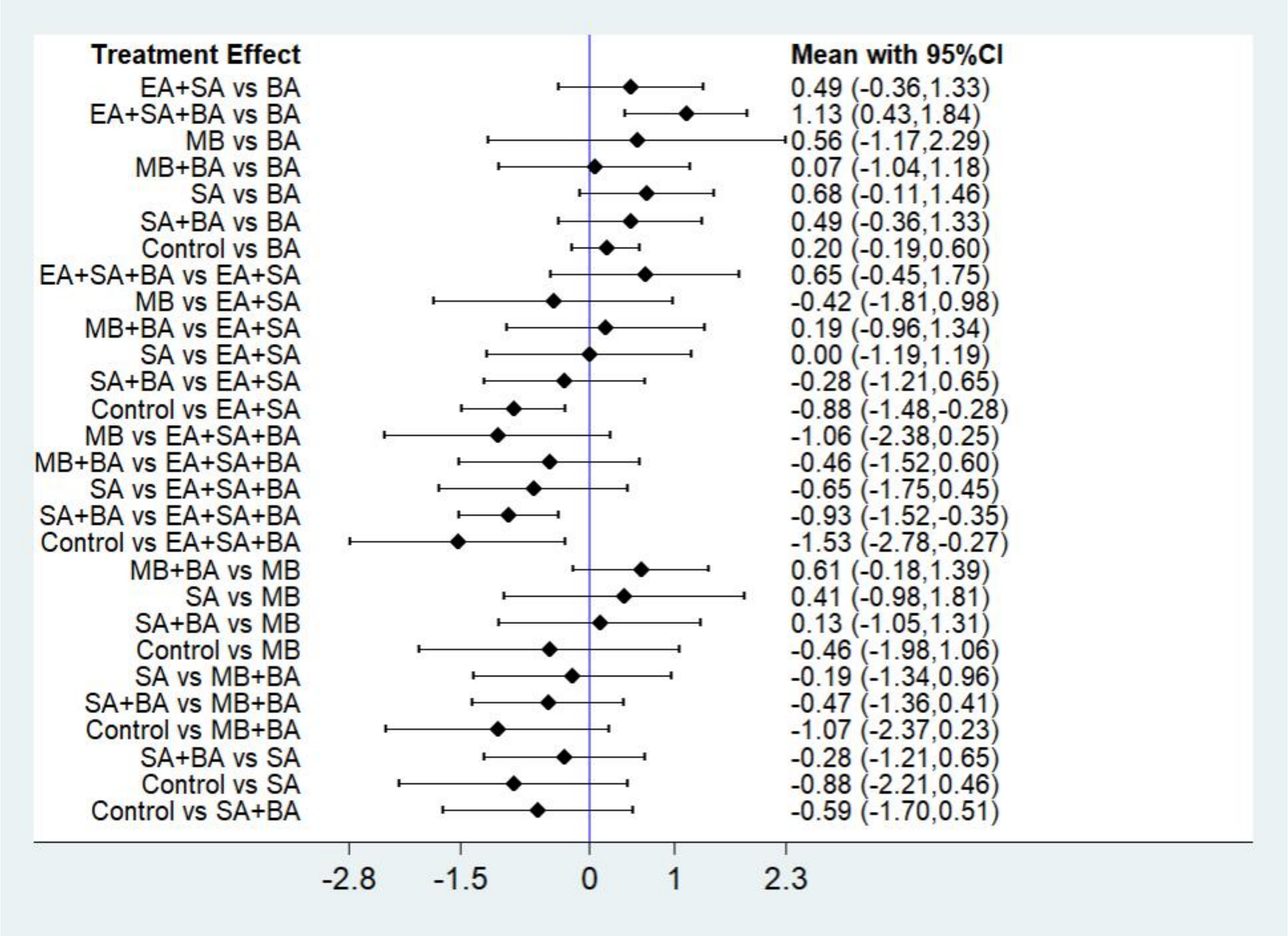

Figure 2. Pair-wise meta-analysis of cognitive function (mid-term)

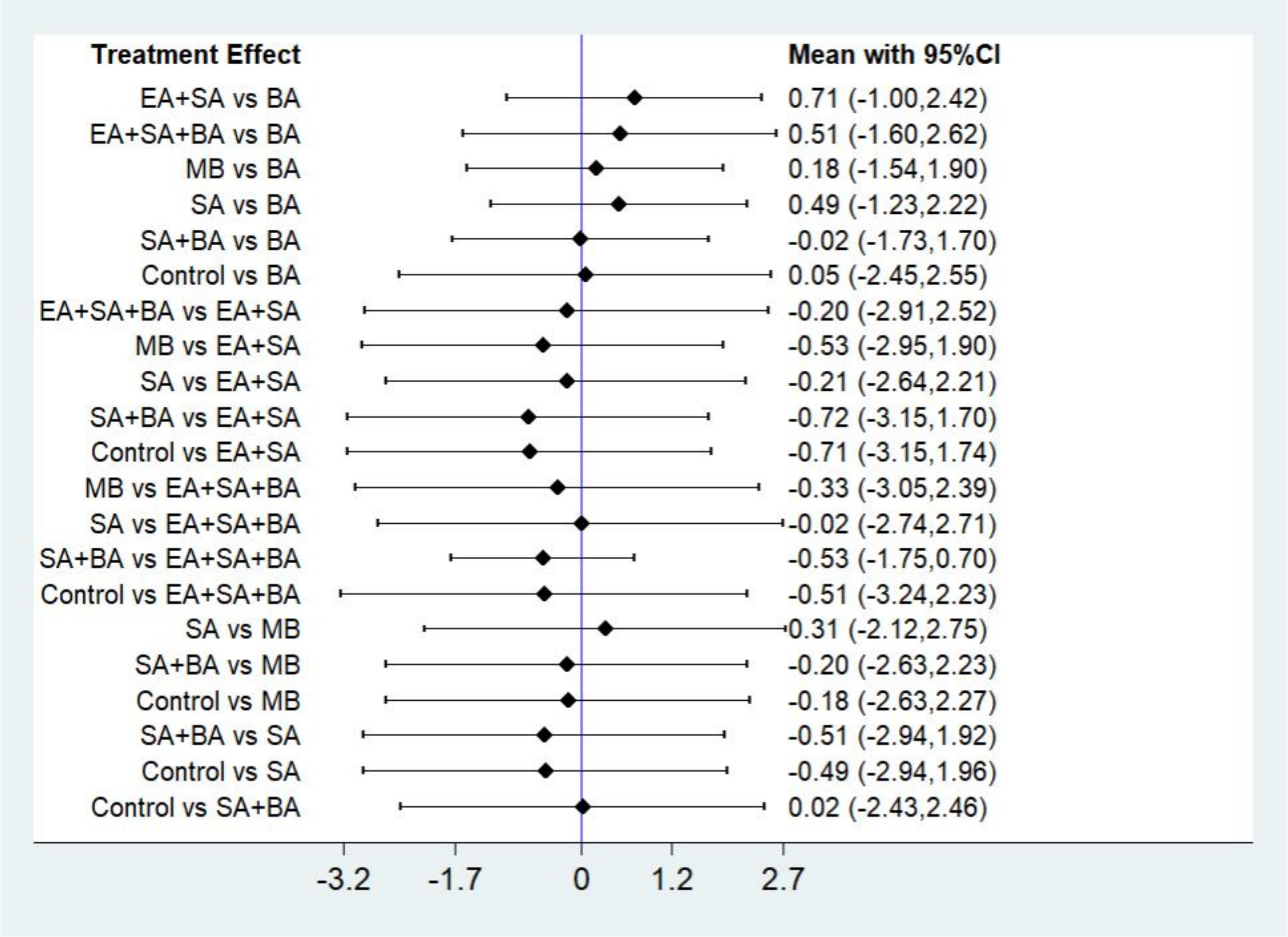

Table 1 Results of network meta-analysis for all possible treatment effects (cognitive function of short-term).

**EA + SA + BA**	.	.	.	0.93 (0.37–1.49)	.	.	.
0.34 (–0.55–1.23)	**MB + BA**	.	0.61 (–0.14–1.35)	.	.	0.68 (–0.07–1.42)	.
0.74 (–0.07–1.55)	0.40 (–0.39–1.19)	**EA + SA**	.	.	.	0.49 (–0.32–1.29)	0.88 (0.30–1.45)
0.80 (–0.12–1.71)	0.46 (–0.18–1.09)	0.06 (–0.73–0.84)	**MB**	.	.	.	1.02 (0.22–1.82)
0.93 (0.37–1.49)	0.59 (–0.10–1.28)	0.19 (–0.40–0.78)	0.13 (–0.59–0.86)	**SA + BA**	.	0.19 (–0.18–0.56)	0.83 (0.24–1.43)
1.02 (0.24–1.80)	0.68 (–0.08–1.44)	0.28 (–0.33–0.89)	0.22 (–0.52–0.97)	0.09 (–0.46–0.64)	**SA**	0.49 (–0.32–1.29)	0.52 (0.07–0.97)
1.17 (0.52–1.82)	0.83 (0.20–1.46)	0.43 (–0.13–0.98)	0.37 (–0.33–1.07)	0.24 (–0.10–0.58)	0.15 (–0.38–0.67)	**BA**	.
1.65 (0.93–2.37)	1.30 (0.61–1.99)	0.90 (0.41–1.39)	0.85 (0.19–1.51)	0.71 (0.26–1.17)	0.62 (0.22–1.03)	0.48 (0.02–0.93)	**Control**

MB + BA, moxibustion with body acupuncture; EA + SA + BA, electroacupuncture with scalp acupuncture with body acupuncture; SA + BA, scalp acupuncture with body acupuncture; EA + SA, electroacupuncture with scalp acupuncture; MB, moxibustion; BA, body acupuncture. The estimates of mean difference of treatments in the columns versus rows are presented in the lower diagonal elements, whereas those of the row treatments vs. column treatments are presented in the upper diagonal elements. "." represents results not reported in the studies.

Table 2 Results of network meta-analysis for all possible treatment effects (cognitive function of mid-term).

**EA + SA + BA**	.	0.53 (–0.63–1.68)	.	.	.	.
0.51 (–1.05–2.07)	**SA**	.	.	.	0.49 (–1.13–2.11)	1.33 (0.40–2.27)
0.53 (–0.63–1.68)	0.02 (–1.03–1.06)	**SA + BA**	.	.	–0.02 (–1.63–1.60)	1.38 (0.66–0.10)
1.07 (–1.14–3.29)	0.56 (–1.36–2.49)	0.55 (–1.34–2.44)	**MB**	.	0.18 (–1.44–1.80)	.
1.05 (–0.37–2.47)	0.54 (–0.42–1.49)	0.52 (–0.30–1.35)	–0.02 (–1.89–1.84)	**EA + SA**	0.71 (–0.90–2.32)	0.66 (0.15–1.17)
1.25 (–0.26–2.77)	0.74 (–0.29–1.78)	0.73 (–0.25–1.71)	0.18 (–1.44–1.80)	0.20 (–0.72–1.13)	**BA**	0.00 (–1.64–1.64)
1.76 (0.42–3.09)	1.25 (0.41–2.09)	1.23 (0.56–1.91)	0.68 (–1.15–2.52)	0.71 (0.21–1.20)	0.50 (–0.36–1.37)	**Control**

EA + SA + BA, electroacupuncture with scalp acupuncture with body acupuncture; SA + BA, scalp acupuncture with body acupuncture; EA + SA, electroacupuncture with scalp acupuncture; MB, moxibustion; BA, body acupuncture. The estimates of mean difference of treatments in the columns versus rows are presented in the lower diagonal elements, whereas those of the row treatments vs. column treatments are presented in the upper diagonal elements. "." represents results not reported in the studies.
